# Parameterization and Application of the General Amber Force Field to Model Fluoro Substituted Furanose Moieties and Nucleosides

**DOI:** 10.3390/molecules27092616

**Published:** 2022-04-19

**Authors:** Diego E. Escalante, Courtney C. Aldrich, David M. Ferguson

**Affiliations:** 1 Department of Medicinal Chemistry, University of Minnesota, Minneapolis, MN 55455, USA; escal005@umn.edu (D.E.E.); aldri015@umn.edu (C.C.A.); 2 Center for Drug Design, University of Minnesota, Minneapolis, MN 55455, USA

**Keywords:** Amber, force field, molecular mechanics, fluorinated, nucleoside, furanose, NMR, sugar pucker

## Abstract

Molecular mechanics force field calculations have historically shown significant limitations in modeling the energetic and conformational interconversions of highly substituted furanose rings. This is primarily due to the gauche effect that is not easily captured using pairwise energy potentials. In this study, we present a refinement to the set of torsional parameters in the General Amber Force Field (*gaff*) used to calculate the potential energy of *mono*, *di-*, and *gem*-fluorinated nucleosides. The parameters were optimized to reproduce the pseudorotation phase angle and relative energies of a diverse set of *mono-* and *di*fluoro substituted furanose ring systems using quantum mechanics umbrella sampling techniques available in the IpolQ engine in the Amber suite of programs. The parameters were developed to be internally consistent with the *gaff* force field and the TIP3P water model. The new set of angle and dihedral parameters and partial charges were validated by comparing the calculated phase angle probability to those obtained from experimental nuclear magnetic resonance experiments.

## 1. Introduction

**Figure 1:**
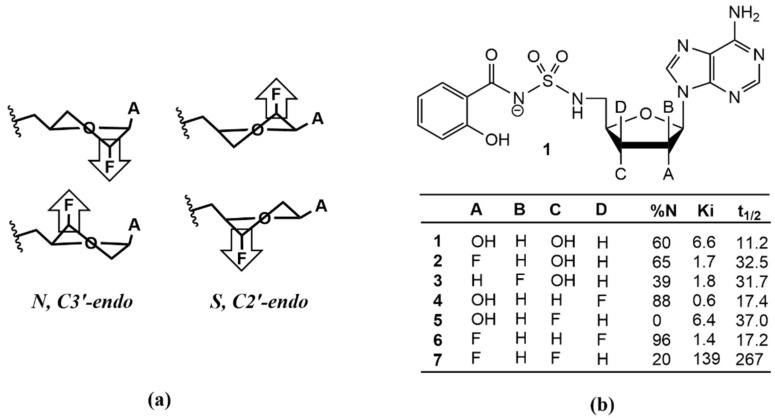
Pucker profile of fluorinated sugars: (**a**) influence of fluorine on ring conformation. The ring pucker is pulled to the side of the most electronegative atom due to the gauche effect; (**b**) ring conformation, enzyme binding, and half-life of fluorinated Sal-AMS derivatives reported in [9].

The selective introduction of fluorine or fluorine-containing functional groups to small organic molecules has proven extremely useful in modifying the physicochemical and pharmacokinetic properties of small drug molecules [1,2,3]. In the field of nucleoside and nucleotide chemistry, fluorine substitutions to the 2′ and 3′ positions of the sugar moiety have been shown to dramatically affect both metabolic stability and biological activity [4,5,6]. This is not only due to the inherent strength of the C–F bond, which is highly resistant to metabolic cleavage but also to the polarizing effect of the fluoro group that influences the sugar pucker angle through the gauche effect [3,6]. In fact, early work on ribo- and arabino-based nucleosides indicated that the position of the fluorine (either up-beta or down-alpha in the 2′ or 3′ position) was a primary determinant of molecular conformation of the sugar moiety, which further influenced the orientation of the base [7,8]. Based on a combination of NMR experiments [1,9,10,11,12,13,14,15] and ab initio molecular orbital calculations [16,17], the preference for a primarily North or South pucker profile was shown to trend with the “up-down” orientation of the fluoro substitution as shown in Figure 1. Of course, the extent of this behavior is driven by the strength of the gauche effect that is known to be correlated to the electronegativity of the substituent. In the case of the 2′-fluoro-2′,3′-dideoxyribose, this effect induces a more Northern pseudorotation angle that is further reinforced by the anomeric effect (for beta-nucleosides) [14]. In contrast, the analogous 2′-fluoro-2′,3′-arabinose derivative adopts a more Southern conformation [10]. It is important to point out that the anomeric effect opposes the gauche effect in this case, resulting in a lower barrier to pseudorotation and a greater distribution of pucker angles and conformational states.

Prior work by our lab has shown fluorination can also have a significant impact on the metabolic stability and biological activity of complex nucleosides [10]. Salicyl adenosine monosulfamate (Sal-AMS) is a selective nanomolar inhibitor of mycobactin biosynthesis that targets the enzyme MbtA [9,10,18,19], responsible for the first and committed biosynthetic step of the mycobactins. This compound is active against whole-cell *Mtb* and in a murine model of acute TB infection but is limited by unfavorable drug disposition properties, including rapid clearance [18,19,20,21]. To address this problem, a series of *mono-* and *di*fluoro substituted furanosyl analogs were synthesized and evaluated using a combination of biological assays and NMR conformational analysis techniques [10]. While the 2′- and 3′-monofluorination offered no improvements to both activity and pharmacokinetic properties, the 2′,3′-difluorodideoxyribose derivative displayed significant gains in half-life (25-fold) and AUC (33-fold) when compared to Sal-AMS as shown in Figure 1b. The improved pharmacokinetic properties, however, come at the cost of binding affinity that is shown to drop by approximately 20-fold. This is most likely due to the failure of this analog to adopt the C3′-endo (North) conformation that is the favored sugar pucker of Sal-AMS in the MbtA binding site. This hypothesis is further supported by the increase in activity noted for the 2′,3′-difluoroxylose derivative. In this case, the gauche and anomeric effects synergize to “lock” the pucker angle in the North and heavily favor the C3′-endo conformation and preferred binding geometry of MbtA.

One of the primary challenges to exploring the relationship between pucker angle, biological activity, and pharmacokinetic parameters in the context of selective fluorination of the sugar moiety is the synthesis. The selective installation of fluoro groups, especially *di-* and *tri-*substitutions can be extremely laborious and in some cases, synthetically insurmountable, leaving large holes in the structure–activity relationship (SAR) plans and strategies. While the influence of 2′- and 3′-monofluoro substitutions on sugar conformation is well established, the effect of multiple substitutions on preference to adopt a more North or South pucker angle is more difficult to predict. It is simply impossible to gauge the balance in gauche and anomeric effects that drive the conformational equilibria when multiple electronegative groups are present. Although ab initio quantum mechanical (QM) calculations have been applied in prior studies to gain insight into the intramolecular forces that determine sugar pucker angles [16,17], methods of this type are computationally taxing and are often used to provide static pictures and relative energies of model structures, typically in vacuo or in implicit solvent [16,17,22,23]. These methods are also not readily extended to evaluate enzyme–ligand interactions and equilibrium properties (including free energies) of macromolecular systems in solution on a large scale [24]. Historically, these limitations have been overcome using molecular dynamic (MD) calculations [22,23,25,26] in which the energy of the system is calculated using molecular mechanics (MM) calculations or hybrid QM/MM techniques in which a subset of atoms are treated using the QM Hamiltonian. Overwhelmingly, the most common method to calculate the physical properties of macromolecular systems is MM calculations, which rely on empirical force fields to model the system using pairwise energy functions [22,23,25,26,27,28,29,30,31,32]. While MM force fields are capable of greatly reducing the computational costs of macromolecular calculations (compared to QM techniques) and are easily extendable, the results are known to depend heavily on the parameterization of the force field [24]. For protein or nucleic acid simulations of standard amino acids, bases, and sugars, the parameter sets are well-vetted and produce excellent static and dynamic physical properties of molecules on all scales [27,28,29,31]. The development of parameters for new entities that are not validated as part of the self-consistent force field (i.e., non-standard molecules, residues, and fragments), however, can be problematic and requires significant effort in fitting the energetics of model systems to experimentally derived data. The process has proven to be quite difficult for highly substituted carbohydrates, including ribosyl moieties and related furanoses [24,33]. This is in part due to the limited structural data available to derive and validate parameter sets for these systems but more so to the inherent limitations of pairwise potential energy functions in capturing the electronic effects that significantly influence the preferred sugar pucker angles.

Although several advances have been made to improve the applicability of force field calculations to carbohydrates, prior studies have not adequately addressed the development of transferrable parameters for modeling fluoro substituted ribosyl moieties and nucleosides. In this study, we tackle this problem using the generalized AMBER force field (*gaff*) as the starting point for development. The approach taken applies well-established techniques and algorithms available through the AMBER suite of programs to generate and optimize atomic charge sets and torsional constants to be consistent with the *gaff* and *ff14sb* force fields. The parameters are initially fit and validated to reproduce ab initio molecular orbital calculated energies and sugar pucker conformations of *mono-* and *di-* substituted fluorofuranose ring systems. A complete analysis of the physical properties calculated using the standard *gaff* assigned parameters and the revised parameters is also presented to gain insight into the limitations of current force field methods in modeling fluoro substituted sugars. Finally, molecular dynamics calculations are applied to model sugar pucker profiles for direct comparisons with experimentally derived structural data of the six fluoros substituted Sal-AMS analogs as well as other fluorinated furanose sugars reported in the literature. The results show the revised parameter set offers significant advantages in the application of *gaff* to the simulation and structural analysis of fluorinated sugar moieties and nucleosides.

## 2. Results and Discussion

**Figure 2:**
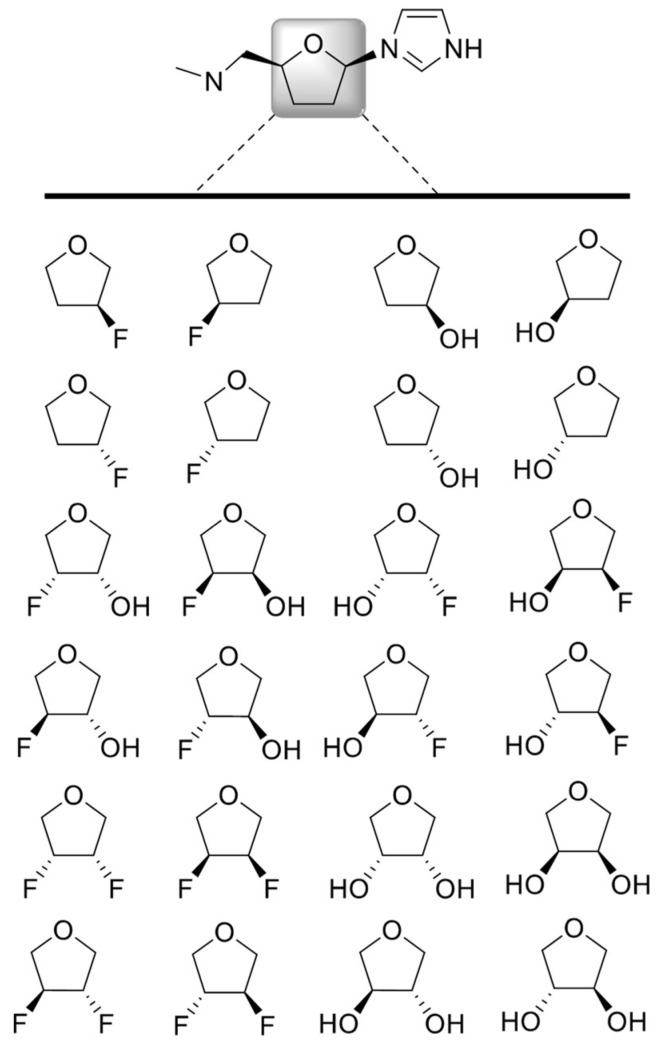
Set of 24 test (T) furanose ring structures used to derive partial charges and parameterize torsional variables.

To evaluate the ability of the *sugar_mod* parameter set to adequately capture the QM energy profiles of the training set test structures, a regression analysis was performed using the standard *gaff* force field parameter set with AM1-BCC partial charges, and the revised *sugar_mod* parameter set with *IpolQ* partial charges. A total of 60,000 structures were generated using the *mdgx* program to sample the pseudorotation space of all the molecules shown in Figure 2. Structures for all molecules in Figure 2 were generated to sample pucker angles at 18-degree intervals between 0 ≤P ≤360. The MM single-point energies for each structure were calculated using *pmemd* with either the AM1-BCC + *gaff* or *IpolQ* + *sugar_mod* parameters. The single-point QM energy was calculated, for the same set of structures as MM, using Gaussian with the B3LYP method, MP2 level, and the *cc*-pVDZ basis set. The modest double polarized *cc*-pVDZ basis set was chosen over higher basis sets to ensure the *IpolQ* + *sugar_mod* parameters are consistent with the *ff14SB* force field for proteins and the TIP3P water model. Furthermore, the level of the quantum theory is not expected to be the limiting factor in improving the force field model, given the fundamental approximations of additivity, fixed partial charges, and harmonic bond and angle vibrations used to develop the parameters [27]. Instead, these limitations are overcome by increasing the conformational diversity of the training set and sampling the entire sugar pucker pseudorotation space.

**Figure 3:**
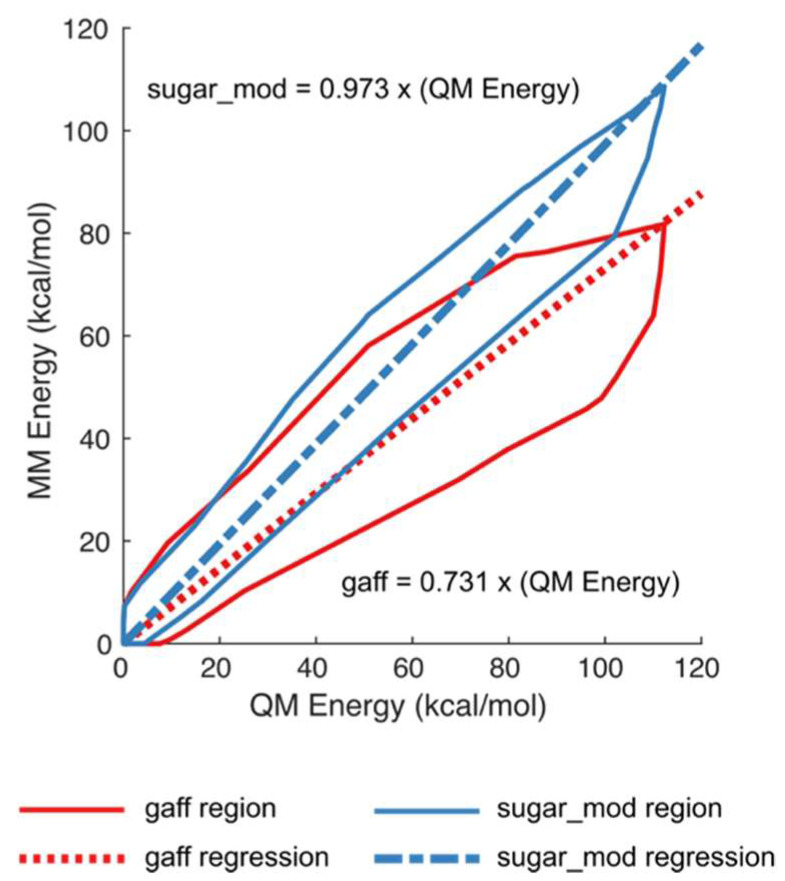
Comparison of molecular mechanics (MM) energies calculated using the *gaff* and *sugar_mod* force field parameters vs. quantum mechanics (QM) energies. The total sample size used was *n* = 47,596 structures. For clarity purposes, only the boundary of all data points is shown rather than individual data points. The red, solid and dashed, lines represent the energies calculated using the *gaff* force field parameters. The blue, solid and dashed, lines represent the energies calculated using the *sugar_mod* force field parameters.

The *gaff* force field parameters tend to underestimate the QM calculated energy by approximately 30%, i.e., *gaff* = (0.731 × QM Energy) with r^2^ = 0.864; Figure 3. On the other hand, the newly parameterized *sugar_mod* force field has an almost 1:1 relationship between MM and QM values, i.e., *sugar_mod* = (0.973 × QM Energy) with r^2^ = 0.976. In addition, the default *gaff* force field parameters underestimate the total potential energies of the ring systems, especially at higher energy values. This is an indication that energy barriers between the sugar puckering conformational phase space are not properly described by the current *gaff* parameters. As a result, transitions between minima energy wells can occur at a higher rate than their true experimental observations, leading to an erroneous characterization of the system. Our results also demonstrate that the *gaff* force field yields a broader distribution of MM energies for sugar puckering structures at QM equipotential values (see Supplemental Figures S1 and S2, and comparison between r^2^ values). The broadening of energy sampling may become particularly problematic when calculating relative binding free energies (ΔΔG) using free energy perturbation (FEP) or thermodynamic integration (TI) methods, since the error of the calculated relative free energy is directly dependent on the standard deviation of the simulation’s observed energy distribution. Consequently, a larger standard deviation results directly in larger errors and reduced confidence in calculated values, once again, highlighting the importance of accurate force field parameters.

**Figure 4:**
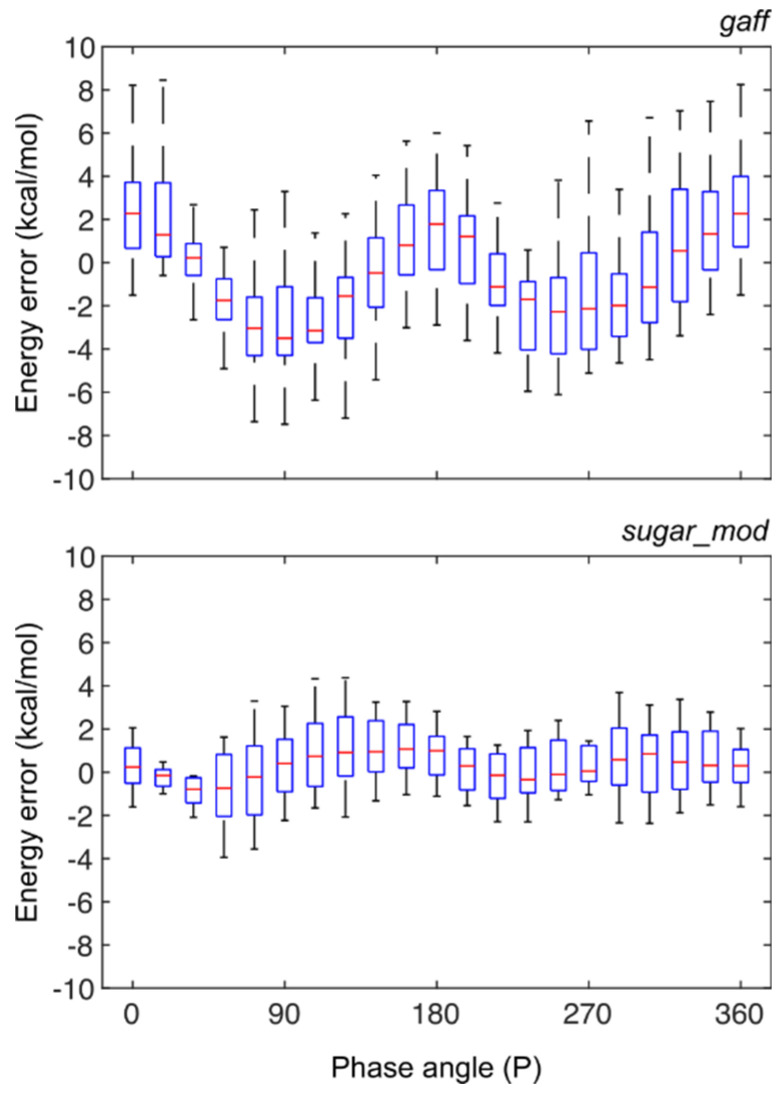
Energy error statistics vs. sugar pucker phase angle (P) for (**top**) *gaff* force field and (**bottom**) *sugar_mod* force field parameters. The energy error was calculated as the difference between the average MM energy in 20 ns simulations at specific *p* values and the respective QM energy; both MM and QM simulations had structures with frozen sugar-heavy atoms. The boxplots show the statistical analysis for structures T = 1–24. On each distribution box, the red central mark indicates the average value (μ), the bottom and top edges of the box indicate one standard deviation (μ ± σ) and the whiskers extend to two standard deviations (μ ±  2σ).

The accuracy of *sugar_mod* force field parameter set was further evaluated through constrained MD simulations of test compounds T = 1–24 to sample conformational energies across the entire pseudorotation cycle. Once again, ring pucker conformations were generated at 18° intervals using the *mdgx* module and subsequently immersed in a periodic box of TIP3P water molecules. Positional harmonic constraints were applied to the heavy atoms of the furanose ring and all systems were equilibrated under *NPT* conditions (300 K and 1 bar) for 20 ns. The minimized QM energy for each ligand at constrained *p* values (E_QM_(P)) was calculated using the MP2 level and the *cc*-pVDZ basis set, and the minimized MM energy was calculated using *pmemd* with the *IpolQ* charges and *sugar_mod* parameters. The *p* value was constrained by freezing the sugar-heavy atoms in place. The average energy error (EE) between the MM and QM energies for all ligands, shown in Figure 4, was calculated using Equations (1) and (2):

(1) $${\mathrm{EE}}_{L}\left(P\right){=E}_{\mathrm{MM},L}\left(P\right)-E_{\mathrm{QM},L}\left(P\right)$$

(2) $${\mathrm{EE}}_{L}\left(P\right){=E}_{\mathrm{MM},L}\left(P\right)-E_{\mathrm{QM},L}\left(P\right)$$

where the subscript L denotes the energy error specific to each individual test model.

The average energy error at a two standard deviation level (i.e., 95% of the total population) for the *gaff* force field yields an error of up to ±8 kcal/mol vs. ±4 kcal/mol obtained from the *sugar_mod* force field, Figure 4. A similar improvement is observed at the one standard deviation level, with errors of ±4 kcal/mol and ±2 kcal/mol for each respective force field. The symmetry breaks for the average error values when using the *gaff* force field (−4.3 < μ < 2.3 kcal/mol) but not for the *sugar_mod* force field (μ = ±0.9 kcal/mol). This further suggests that the *gaff* force field tends to underestimate the energy of a *mono*- or *di*-substituted sugar molecule with a fluorine atom in the C2′ or C3′ positions. In Figure S3 we present the statistical energy average for each *p* value for compounds T = 1–24 with each molecule parameterized using the *gaff* and *sugar_mod* force fields.

**Figure 5:**
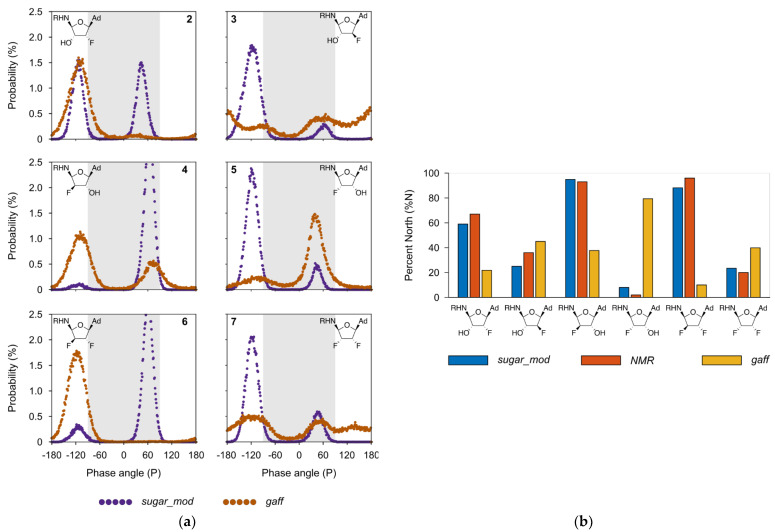
Comparison of puckering between *gaff* and *sugar_mod* force fields for nucleosides **2**–**7**: (**a**) Percent probability of phase angles for six fluorinated and hydroxylated Sal-AMS analogs calculated from unrestrained MD simulations using the *IpolQ+sugar_mod* (purple) and AM1-BCC + *gaff* parameters (brown). The light grey shaded region shows the North configuration region, i.e., −90≤P≤90; (**b**) Comparison of total percent North (% N) probability for the six fluorinated and hydroxylated Sal-AMS analogs calculated from unrestrained MD simulations using the *IpolQ + sugar_mod* (blue) and AM1-BCC + *gaff* parameters (yellow) compared to the literature experimental NMR values [9].

As a final measure of applicability of the new parameter set to accurately model *mono*- and *di*-fluorinated furanose ring systems, the pucker angles, and corresponding energies of compounds **2**–**7** were evaluated using unrestrained MD simulations. Most experiments involving sugar-containing molecules report the ring configuration using the single parameter percent North (% N) or percent South (% S). However, the sugar ring system is a dynamic equilibrium between all possible pseudorotation and amplitude angles [34]. Therefore, we simulated the behavior of the six fluoro and hydroxylated analogs of Sal-AMS shown in Figure 1 through 200 ns of unrestrained MD simulations using both the *gaff* and the *sugar_mod* force field parameters. The calculated probabilities, shown in Figure 5a, were derived based on the simulation time spent at each ring puckering configuration angle. The data show that there is a remarkable difference between the configurations obtained using each of the two different force field parameters.

The simulations show that the *gaff* force field parameters tend to favor either the North (N) *or* South (S) configuration, panels **2**, **4**, **5,** and **6** of Figure 5a. There are certain cases such as the arabino-2′,3′-OH F (**3**) and ribo-2′,3′-FF (**7**) configurations where the *gaff* results suggest there is no clear preference and there is an almost even distribution of all the puckering angles. However, NMR experimental data [10] has demonstrated that there is a predominantly South preference for both **3** and **7**. This shows that in fact, the *gaff* force field is not able to properly capture the behavior of the ring puckering for all cases and that a reparameterization of certain force field terms was warranted. On the other hand, the simulation results using the *sugar_mod* parameters show that in all the tested cases there are clear peaks at either the North or South configurations. The data obtained from the *sugar_mod* simulations show that the C2′ position of the sugar ring tends to have the greatest effect on the conformational position of the pucker. When the fluoro or hydroxyl substitution follows the 2′-endo configurations (i.e., **2**, **3**, **5,** and **7**) the sugar pucker exhibits a Southernly configuration, and the 2′-exo configurations (i.e., **4** and **6**) exhibit a Northerly configuration. Visual inspection of the simulations revealed that the reason for this behavior is the weak electrostatic interaction that occurs between the C2′ substituent (i.e., fluoro or hydroxyl) with an oxygen atom of the sulfamoyl moiety in Sal-AMS and its analogs.

To complete the validation of puckering parameters, we calculated the total percent North (% N) configuration, as the integration between −90≤P≤90 degrees and compared them to the calculated values from experimental NMR data [10]. The wide range of angles chosen, as opposed to the more restricted definition that the North configuration is −18≤P≤18, was due to the way that programs such as PSEUROT use NMR J-constants to calculate the % N value [34,35]. These programs assume there is an N/S dynamic equilibrium for the sugar ring and derives the mole fraction of the ring pucker populating *each hemisphere* of the pseudorotational cycle [36]. The comparison between calculated % N for *gaff* and *sugar_mod* vs. NMR data is shown in Figure 5b.

## 3. Materials and Methods

### 3.1. Structure Generation

A total of 24 test structures (T = 1–24), Figure 2, were constructed using the Schrodinger structure builder tool. Next, a custom PyMOL script was used to translate the sugar ring heavy atoms, i.e., C1′–C4′ and O4′, so that the Cartesian coordinates of the heavy atoms would represent a specific pseudorotation angle (P) based on the Altona and Sudaralingam method [37]. For each molecule T, 20 structures were generated to cover the entire pseudorotation space 0≤P<360 at 18° intervals. This resulted in a total of 480 initial structures (i.e., 20 pseudorotation conformations per each T structure). Using MacroModel, all of the initial structures were subjected to a Polak-Ribiere Conjugate Gradient minimization with restrained sugar-heavy atoms. The set of minimized structures was used as the starting point for subsequent structure generations using *mdgx*.

### 3.2. Calculation of New Parameters

**Figure 6:**
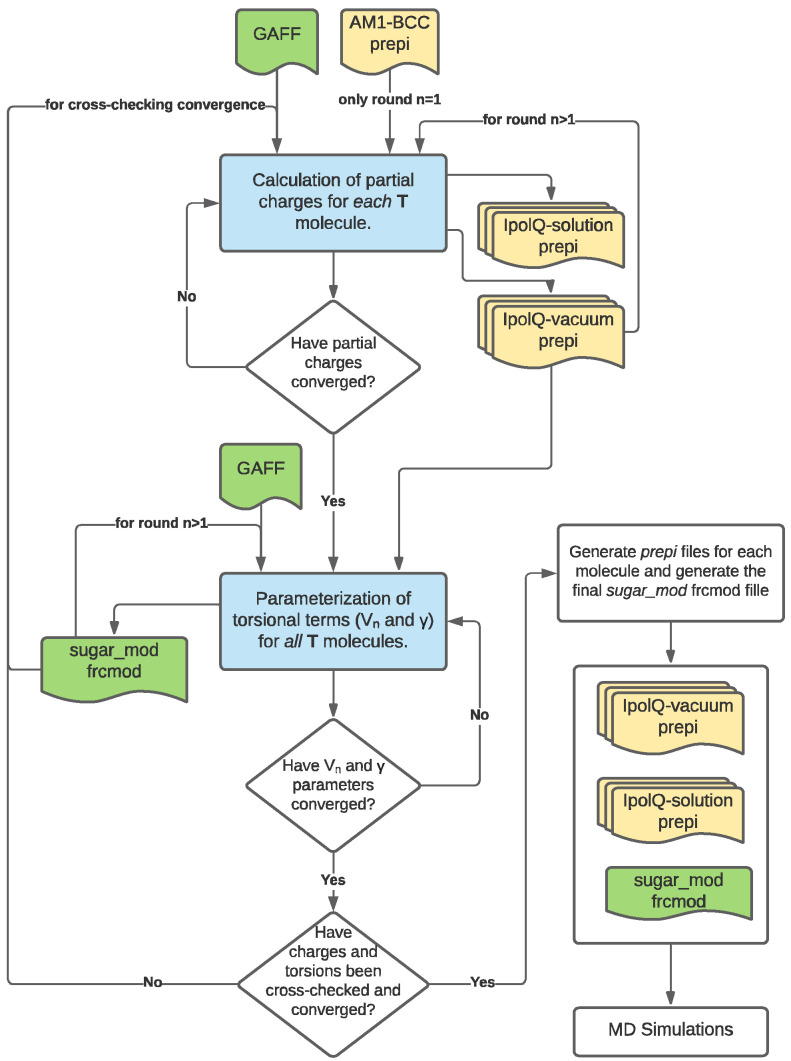
Workflow used to calculate implicitly polarized and gas-phase partial charge sets for each molecule, and, the *sugar_mod* frcmod file containing the reparameterization of $V_{n}$ and γ for the dihedrals of interest. The processes in blue have been explained in detail in Section 3.2.1 and Section 3.2.2 as well as Figure S4. The green and yellow files are force field and *prepi* files, respectively.

The process to calculate the new torsional parameters was divided into two parts: (1) the calculation of new partial atomic charge sets for each molecule; and (2) the parameterization of the torsion parameters $V_{n}$ and γ for the dihedrals of interest. The workflow of the process has been summarized in Figure 6 and explained in greater detail in Section 3.2.1 and Section 3.2.2 along with Figure S4.

#### 3.2.1. Partial Atomic Charges

The *IpolQ* function of the *mdgx* program [33] was used to generate 8 random poses for each of the 420 structures in the starting point set (i.e., 8 structures for each pseudorotation phase angle window of 18°). The set of structures obtained was reduced using a pair-wise culling process in which any structure with a root-mean-squared deviation less than 1 Å to any other molecule was removed. The final set of structures used to calculate the partial charges was comprised of ~64 unique molecules for each model system. Initial charges were obtained using *antechamber* and the default AM1-BCC method. This was followed by an initial assignment of force field parameters using the General Amber Force Field (*gaff*). The structures were subsequently immersed in a TIP3P water box with an 8 Å buffer zone and subjected to a minimization, heating, and equilibration protocol using Amber’s *pmemd* engine (with the solute frozen in place). First, minimization was carried out using 1000 steps of a steepest-descent gradient. Next, the system was heated from an initial temperature of 0 K up to 300 K, using an *NTV* ensemble over a total of 20,000 steps. Finally, the water molecules were equilibrated to a pressure of 1bar, using an *NPT* ensemble over a total of 30,000 steps.

Once all the structures had been prepared, Amber’s *mdgx* engine was used to generate submission scripts for Gaussian to calculate grids containing the electrostatic potential due to the wave function. The grids were calculated both *in vacuo* and in solution at the MP2 level using a cc-pVDZ basis set. Finally, the resulting grids were used to fit the electrostatic potential using the *IPolQ* procedure of *mdgx* and generate a set of partial charges for each test molecule. The calculated set was compared to the previous charge set (i.e., AM1-BCC for the first iteration). If all partial charges varied less than 5% from their previous value the set was determined to be converged. Otherwise, the procedure to calculate partial charges was iterated until convergence was achieved. Each new iteration used the same set of structures along with the previous *IpolQ*-vacuum *prepi* file to seed the next step of partial charges calculations. It is important to note that the AM1-BCC partial charges were only used as a starting point for the first iteration, all the following iteration steps (i.e., *i* > 2) used the previous iteration set of partial charges. This workflow for the procedure is shown in Figure S4.

#### 3.2.2. Torsional Parameters

The *parmchk2* utility in Amber was used to identify all potential combinations of atoms comprising the dihedral angles required to define the 24 test structures listed in Figure 2. A total of 65 unique combinations were obtained (listed in Table S1). However, since we were only interested in parameterizing those containing fluoro and hydroxyl atom types only seven dihedral angles were fitted, Table 1.

The fitting procedure was initiated by generating a total of 2480 structures for each of the 24 furanose ring templates using the *mdgx* structure generator, i.e., 124 random structures for each pseudorotation angle. Unlike the set of structures used for the calculation of charge sets, none of the configurations generated at this stage were removed from the set. All structures were kept since it has been shown that fitting a high dimensional space, i.e., multiple dihedral and angle force field parameters requires a thorough sampling of the configurational space to properly parameterize the entire accessible space [33]. For the first iteration of torsion parameter fitting, each structure (i.e., all 59,520 structures) was subsequently assigned the previously calculated in vacuo *IpolQ* charges as well as *gaff* default force field parameters. Once all the subset structures had been prepared, Amber’s *mdgx* engine was used to generate submission scripts for Gaussian to calculate single point energies for all structures at the MP2 level and cc-pVDZ basis set. Next, *mdgx* was used to calculate the residual energy, i.e., the difference between QM and MM. The residual energy, shown in Equation (3), was minimized by *mdgx* through a multidimensional nonlinear least squared procedure to freely fit the value of $V_{n}$ and a constrained value of γ=0 or γ=180. The remaining terms in Equation (3) (force constants, bond lengths and angles, and van der Walls radii and polarizability) were not modified from the published *gaff* values.

(3) $$E_{res}=E_{\mathrm{QM}}-\underset{\mathrm{bonds}}{\sum }K_{b}{\left(b-b_{0}\right)}^{2}+\underset{\mathrm{angles}}{\sum }K_{\theta }{\left(\theta -\theta_{0}\right)}^{2}+\;\sum_{\mathrm{nonb}}\;ij\frac{A_{ij}}{r_{ij}^{12}}-\frac{B}{r_{ij}^{6}}+\frac{q_{i}q_{j}}{r_{ij\;}}+\sum_{\mathrm{dihedrals}}^{7}\sum_{n=1}^{3}V_{n}\left[1+\cos\left(n\phi -\gamma \right)\right],$$

The newly fitted parameters were compared to the previous iteration force field values (i.e., *gaff* for the first iteration). If all $V_{n}$ values varied less than 5% from its previous value and all γ remained the same, the new set of parameters were determined to be converged. Otherwise, the procedure to parameterize the fluoro and hydroxy dihedral angles was iterated until convergence was achieved. (Note: Since one of the constraints given to *mdgx* was to use a high harmonic restraint on angle stiffness constant (*arst*) the equilibrium angle ($\theta_{0}$) was not included in the fitting procedure.) We would like to emphasize that the original *gaff* parameters were only used as a starting point for the first iteration, all the following iteration steps (i.e., *i* > 2) used the fitted values in the previous iteration. This procedure is summarized in the flowchart shown in Figure S4. The final stage in the re-parameterization was to check that the new parameters did not change the calculated partial charge sets. Therefore, a full iteration of both partial charge set calculations and torsional parameterization was performed. Once convergence was achieved, the new parameters were assembled into a frcmod file called *sugar*_*mod*, which can be found in the supplemental information (Table S3). The *prepi* files containing the final partial atomic charges and corresponding structures are given in Figure S5 and Tables S4–S27.

### 3.3. Molecular Dynamic Simulations

Molecular Dynamic (MD) simulations were carried out using the *pmemd* function of the AMBER 18 software package. Ligand structures were preprocessed to assign either AM1-BCC or the calculated *IpolQ* partial charges. The ligands were then processed using *tLeap* to assign *gaff* force field parameters as well as the calculated *sugar_mod* force field modification (*frcmod*) parameters. All ligands were submerged in a cubic TIP3P water box with a 10 Å buffer region. This was followed by a step-wise *NVT* heating procedure in which the system temperature was gradually ramped from 0 to 300 K over 15,000 steps and subsequently relaxed over 5000 steps in which the average temperature was kept constant at 300 K using the weak-coupling algorithm. During both stages of heating, the position of all non-solvent atoms was restrained with a harmonic potential force constant of 100 kcal/mol Å. After heating, the systems were equilibrated to a pressure of 1 bar using the *NPT* ensemble for 20,000 steps with all non-solvent atoms restrained with a harmonic potential with a force constant of 100 kcal/mol Å. This was followed by a relaxation stage. This relaxation stage varied depending on whether the production run would be for: (i) unrestrained simulations, or (ii) simulations where the sugar ring (i.e., the five heavy atoms C1′–C4′ and O4′) had to be constrained to a particular pseudorotation angle. In the first case, all non-solvent atoms were restrained with a harmonic potential with a force constant of 0.5 kcal/mol Å. For the second case, the heavy sugar atoms were restrained with a harmonic potential with a force constant of 100 kcal/mol Å to maintain the desired pseudorotation angle, and the rest of the non-solvent atoms were restrained with a harmonic potential with a force constant of 0.5 kcal/mol Å. The minimization, heating, and equilibration stages were all carried out using the PMEMD.MPI function. All production runs were carried out using the *NPT* ensemble and PMEMD.CUDA function. For unrestrained simulations, the total calculation time was 200 ns per ligand. On the other hand, the phase analysis simulations required that the sugar-heavy atoms be restrained with a harmonic potential with a force constant of 100 kcal/mol Å. This type of simulation was run for 20 ns per pseudorotation angle per ligand (i.e., 360 ns per ligand).

## 4. Conclusions

The primary goal of this work was to develop a parameter set for simulating fluoro substituted nucleosides using the tools available in the AMBER suite of programs. The approach followed standard procedures and methods to derive partial charges and torsional parameters to be consistent with the *gaff* force field and standard parameter sets. Using 24 *mono-* and *di*-fluoro substituted furanose ring systems, the parameters were fit to reproduce ab initio molecular orbital energies of sugar pucker conformations spanning the entire pseudorotation cycle. The refinement process ensured that both high and low energy conformations were adequately sampled to reproduce the energies barriers to pucker interconversions. The results show the refined parameter set reproduced the sugar pucker energies calculated using the cc-pVDZ basis set at the MP2 level to near linearity (RMS = 0.973). The refined parameter set was also applied to predict the pucker preferences for a set of mono- and difluoro sugar substituted nucleosides for comparison with experimentally determine NMR structural data. The results show the calculated percent North (*%* N) pucker probabilities matched the NMR derived values within an 8% error. Direct comparisons were also performed to evaluate the performance of the default *gaff* parameter set assigned by *TLeaP*. In general, the standard parameters were found to underestimate the energy barriers to pseudorotation, which in turn caused the energy profiles to be flatter. This was shown to produce more variance in the pucker angles and more rapid interconversions. Essentially, the ring is too flexible. In addition, the default parameters failed to identify the dominant sugar pucker conformation of several nucleosides as shown in Figure 5b. It is therefore reasonable to conclude that the default *gaff* assigned parameters are not suitable for modeling fluorinated sugar rings.

Finally, to aid in transferability, the parameters were purposely developed to be consistent with the *gaff* force field and associated suite of programs. This should allow the parameter set to be readily extended to model other sugar ring systems containing polar substitutions with minimal refinements to the seven dihedral angles reported in Table 1. Given the increasing use of highly substituted sugars (especially halogenated moieties) in the design of nucleosides and carbohydrate building blocks applied in drug discovery, we expect the new parameter set and techniques described will find widespread applications in modeling the physical and structural properties of a variety of drug molecules as well as novel RNA and DNA oligomers.

## Figures and Tables

**Table 1:** List of dihedral angles fitted with atom types assigned by *tLeap*.

Dihedral Angle			
c3	c3	c3	oh
c3	c3	c3	f
c3	c3	oh	ho
f	c3	c3	f
f	c3	c3	oh
f	c3	c3	os
oh	c3	c3	oh

## Data Availability

Not applicable.

## Supplementary Materials

The following supporting information can be downloaded at: https://www.mdpi.com/article/10.3390/molecules27092616/s1, Figure S1: Comparison of total energy between AM1-BCC + *gaff* molecular mechanics (MM) and quantum mechanics (QM) calculations; Figure S2: Comparison of total energy between *IPolQ* + *sugar_mod* molecular mechanics (MM) and quantum mechanics (QM) calculations; Figure S3: Energy profiles with sugar-heavy atoms frozen at different puckering phase; Figure S4: Partial charge calculation and torsion angle reparameterization workflow; Figure S5: Model structures descriptions; Table S1: List of all torsions identified by the *parmchk2* utility; Table S2: List of all angles identified by the *parmchk2* utility. Table S3: Torsional force field parameter set. Table S4: prepi file for test molecule **1**; Table S5: prepi file for test molecule **2**; Table S6: prepi file for test molecule **3**; Table S7: prepi file for test molecule **4**; Table S8: prepi file for test molecule **5**; Table S9: prepi file for test molecule **6**; Table S10: prepi file for test molecule **7**; Table S11: prepi file for test molecule **8**; Table S12: prepi file for test molecule **9**; Table S13: prepi file for test molecule **10**; Table S14: prepi file for test molecule **11**; Table S15: prepi file for test molecule **12**; Table S16: prepi file for test molecule **13**; Table S17: prepi file for test molecule **14**; Table S18: prepi file for test molecule **15**; Table S19: prepi file for test molecule **16**; Table S20: prepi file for test molecule **17**; Table S21: prepi file for test molecule **18**; Table S22: prepi file for test molecule **19**; Table S23: prepi file for test molecule **20**; Table S24: prepi file for test molecule **21**; Table S25: prepi file for test molecule **22**; Table S26: prepi file for test molecule **23**; Table S27: prepi file for test molecule **24**.
