# Supplementary material for: Parameterization and Application of the General Amber Force Field to Model Fluoro Substituted Furanose Moieties and Nucleosides
Source: Molecules. 2022 Apr 19;27(9):2616. doi: 10.3390/molecules27092616 (PMC9101125; doi:10.3390/molecules27092616)
Supplement: Supplementary file 1 [file molecules-27-02616-s001.zip › molecules-1644730-supplementary.pdf]

Supplemental Information

Parameterization and Application of the General Amber Force Field to Model Fluro Substituted Furanose Moieties and Nucleosides

Diego E. Escalante [1], Courtney C. Aldrich [1] and David M. Ferguson [1,2,*]

[1] Department of Medicinal Chemistry, University of Minnesota, Minneapolis, MN 55455, USA; escal005@umn.edu (D.E.E.); aldri015@umn.edu (C.C.A.)
[2] Center for Drug Design, University of Minnesota, Minneapolis, MN 55455, USA
* Correspondence: ferguson@umn.edu

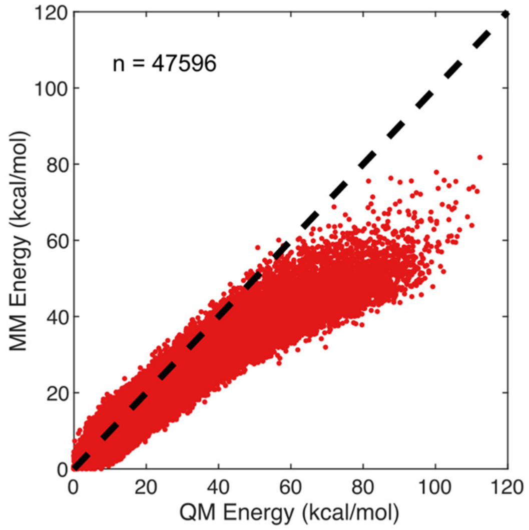

Figure S1. Comparison of total energy between AM1-BCC+*gaff* molecular mechanics (MM) and quantum mechanics (QM) calculations. The total MM energy was calculated using the standard *gaff* force field parameters. The total QM energy was calculated at the MP2 level using the cc-PvDZ basis set. The total sample included 47,596 structures, i.e. an average 1983 structures per configuration (**1–24**). The black dashed line indicates a 1:1 linear relationship between the two data sets.

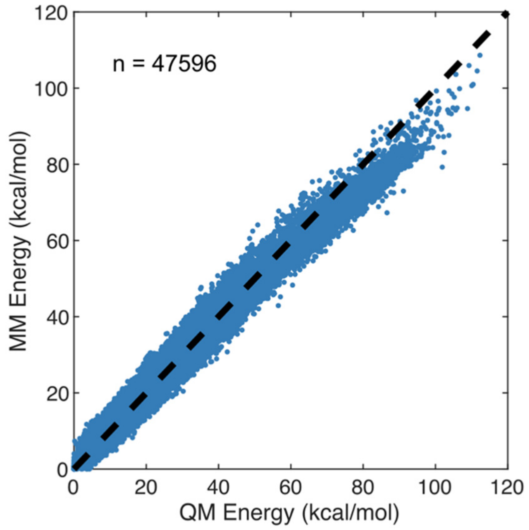

Figure S2. Comparison of total energy between *IPolQ+sugar_mod* molecular mechanics (MM) and quantum mechanics (QM) calculations. The total MM energy was calculated using the fitted parameters calculated by *mdgx*, referred to as the *sugar_mod* force field parameters. The total QM energy was calculated at the MP2 level using the cc-PvDZ basis set. The total sample included 47,596 structures, i.e. an average 1983 structures per configuration (**1–24**). The black dashed line indicates a 1:1 linear relationship between the two data sets.

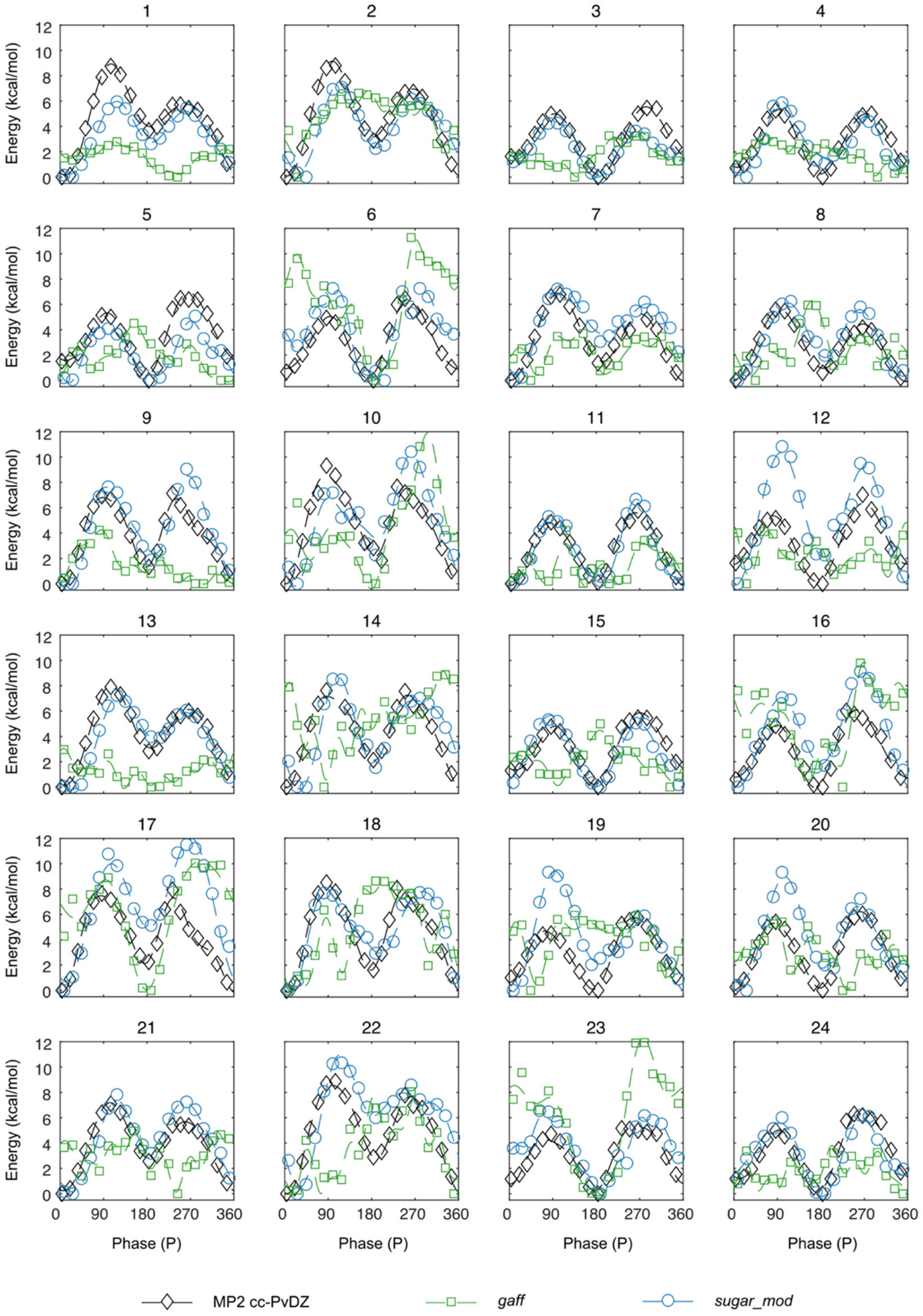

Figure S3. Energy profiles with sugar heavy atoms frozen at different puckering phase angles (P). Quantum mechanical energies were calculated at the MP2 level using the cc-PvDZ basis set and are shown as black diamonds. The molecular mechanical energies shown in this figure represent the statistical average for each P value during a 20 ns simulation (i.e. a total simulation time of 400 ns for each test structure). The results for the *gaff* and *sugar_mod* force fields are shown as green squares and blue circles, respectively.

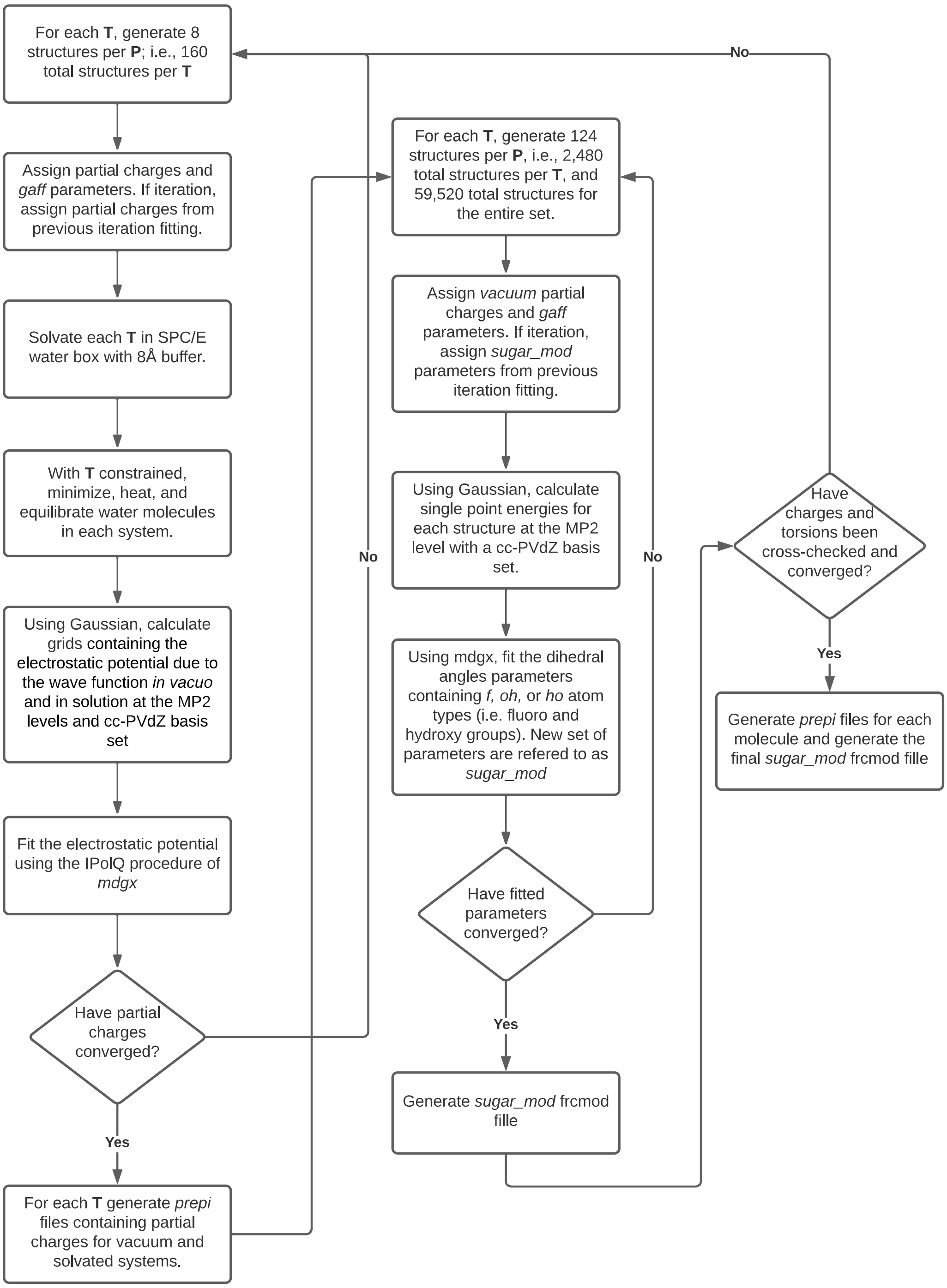

Figure S4. Partial charge calculation and torsion angle reparameterization workflow. *(left)* Workflow used to calculate implicitly polarized and gas-phase partial charge sets for *each* molecule, and, (*right*) workflow used to generate the *sugar_mod* frcmod file containing the reparameterization of V_n and γ for the dihedrals of interest.

Table S1. List of all torsions identified by the *parmchk2* utility.

Torsion parameters			
c3-c3-c3-c3	cc-cd-nd-cc	h1-c3-c3-na	h4-cc-na-cc
c3-c3-c3-f	cd-cc-na-c3	h1-c3-c3-oh	h4-cd-nd-cc
c3-c3-c3-h1	cd-cc-na-cc	h1-c3-c3-os	h5-cc-na-c3
c3-c3-c3-h2	f-c3-c3-f	h1-c3-n3-c3	h5-cc-na-cc
c3-c3-c3-hc	f-c3-c3-h1	h1-c3-n3-hn	h5-cc-nd-cd
c3-c3-c3-n3	f-c3-c3-h2	h1-c3-oh-ho	hc-c3-c3-na
c3-c3-c3-na	f-c3-c3-hc	h1-c3-os-c3	hc-c3-c3-oh
c3-c3-c3-oh	f-c3-c3-na	h2-c3-c3-hc	hc-c3-c3-os
c3-c3-c3-os	f-c3-c3-oh	h2-c3-c3-oh	n3-c3-c3-os
c3-c3-n3-c3	f-c3-c3-os	h2-c3-na-cc	na-c3-c3-oh
c3-c3-n3-hn	h1-c3-c3-h1	h2-c3-os-c3	na-c3-os-c3
c3-c3-na-cc	h1-c3-c3-h2	h4-cc-cd-h4	na-cc-cd-h4
c3-c3-oh-ho	h1-c3-c3-hc	h4-cc-cd-nd	na-cc-cd-nd
c3-c3-os-c3	h1-c3-c3-n3	h4-cc-na-c3	na-cc-nd-cd
nd-cc-na-cc	oh-c3-c3-oh	oh-c3-c3-os	nd-cc-na-c3
os-c3-na-cc			

Table S2. List of all angles identified by the *parmchk2* utility.

Angle parameters		
c3-c3-c3	c3-oh-ho	h1-c3-os
c3-c3-f	c3-os-c3	h2-c3-na
c3-c3-h1	cc-cd-h4	h2-c3-os
c3-c3-h2	cc-cd-nd	h4-cc-na
c3-c3-hc	cc-na-cc	h4-cd-nd
c3-c3-n3	cc-nd-cd	h5-cc-na
c3-c3-na	cd-cc-h4	h5-cc-nd
c3-c3-oh	cd-cc-na	hc-c3-hc
c3-c3-os	f-c3-h1	na-c3-os
c3-n3-c3	h1-c3-h1	na-cc-nd
c3-n3-hn	h1-c3-n3	h1-c3-oh
c3-na-cc		

Table S3. Refined set of torsional parameters used to calculate the potential energy of mono, di-, and gem-fluorinated nucleosides in combination with the AMBER General Force Field (*gaff*). For further information on term usage refer to Section 14.1.4 of the AMBER 18 user manual.

Dihedral Definition	Divider	Barrier term (V_n)	Phase (γ)	Periodicity (n)
c3-c3-c3-os	1	0.070	180.000	-3.000
c3-c3-c3-os	1	0.031	180.000	-2.000
c3-c3-c3-os	1	0.008	180.000	1.000
h1-c3-c3-os	1	0.237	180.000	-3.000
h1-c3-c3-os	1	0.427	180.000	-2.000
h1-c3-c3-os	1	0.042	0.000	1.000
c3-c3-c3-c3	1	0.148	180.000	-3.000
c3-c3-c3-c3	1	0.137	0.000	-2.000
c3-c3-c3-c3	1	0.159	180.000	1.000
c3-c3-c3-hc	1	0.449	0.000	-3.000
c3-c3-c3-hc	1	0.679	180.000	-2.000
c3-c3-c3-hc	1	0.598	180.000	1.000
c3-c3-os-c3	1	0.145	180.000	-3.000
c3-c3-os-c3	1	0.298	0.000	-2.000
c3-c3-os-c3	1	0.212	0.000	1.000
c3-c3-c3-h1	1	0.648	0.000	-3.000
c3-c3-c3-h1	1	0.434	180.000	-2.000
c3-c3-c3-h1	1	0.397	0.000	1.000
h1-c3-c3-h1	1	0.393	0.000	-3.000
h1-c3-c3-h1	1	0.150	180.000	-2.000
h1-c3-c3-h1	1	0.414	180.000	1.000
h2-c3-os-c3	1	0.307	0.000	-3.000
h2-c3-os-c3	1	0.347	180.000	-2.000
h2-c3-os-c3	1	0.108	0.000	1.000
na-c3-os-c3	1	0.461	180.000	-3.000
na-c3-os-c3	1	0.248	0.000	-2.000
na-c3-os-c3	1	0.058	180.000	1.000
c3-c3-c3-h2	1	0.842	180.000	-3.000
c3-c3-c3-h2	1	0.651	0.000	-2.000
c3-c3-c3-h2	1	0.215	180.000	1.000
c3-c3-c3-na	1	0.667	180.000	-3.000
c3-c3-c3-na	1	0.127	0.000	-2.000
c3-c3-c3-na	1	0.104	0.000	1.000
hc-c3-c3-os	1	0.100	0.000	-3.000
hc-c3-c3-os	1	0.032	0.000	-2.000
hc-c3-c3-os	1	0.945	180.000	1.000
h2-c3-c3-hc	1	0.153	180.000	-3.000
h2-c3-c3-hc	1	0.603	0.000	-2.000
h2-c3-c3-hc	1	0.644	0.000	1.000
hc-c3-c3-na	1	0.742	0.000	-3.000

hc-c3-c3-na	1	0.675	0.000	-2.000
hc-c3-c3-na	1	2.274	0.000	1.000
h1-c3-c3-hc	1	0.320	0.000	-3.000
h1-c3-c3-hc	1	0.291	180.000	-2.000
h1-c3-c3-hc	1	1.364	0.000	1.000
h1-c3-os-c3	1	0.031	180.000	-3.000
h1-c3-os-c3	1	0.050	0.000	-2.000
h1-c3-os-c3	1	0.083	180.000	1.000
c3-c3-c3-f	1	0.280	180.000	-3.000
c3-c3-c3-f	1	0.521	0.000	-2.000
c3-c3-c3-f	1	1.821	180.000	1.000
f-c3-c3-h1	1	0.936	0.000	-3.000
f-c3-c3-h1	1	0.616	180.000	-2.000
f-c3-c3-h1	1	1.237	0.000	1.000
f-c3-c3-f	1	0.634	180.000	-3.000
f-c3-c3-f	1	1.502	0.000	-2.000
f-c3-c3-f	1	3.450	180.000	1.000
f-c3-c3-os	1	0.174	0.000	-3.000
f-c3-c3-os	1	0.800	0.000	-2.000
f-c3-c3-os	1	0.655	180.000	1.000
f-c3-c3-h2	1	0.134	180.000	-3.000
f-c3-c3-h2	1	0.837	0.000	-2.000
f-c3-c3-h2	1	0.357	180.000	1.000
f-c3-c3-na	1	0.552	180.000	-3.000
f-c3-c3-na	1	0.003	0.000	-2.000
f-c3-c3-na	1	0.199	0.000	1.000
h1-c3-c3-h2	1	1.598	0.000	-3.000
h1-c3-c3-h2	1	0.089	0.000	-2.000
h1-c3-c3-h2	1	0.080	0.000	1.000
h1-c3-c3-na	1	1.801	0.000	-3.000
h1-c3-c3-na	1	0.597	180.000	-2.000
h1-c3-c3-na	1	0.762	0.000	1.000
c3-c3-c3-oh	1	0.051	180.000	-3.000
c3-c3-c3-oh	1	0.732	0.000	-2.000
c3-c3-c3-oh	1	1.871	180.000	1.000
c3-c3-oh-ho	1	0.190	0.000	-3.000
c3-c3-oh-ho	1	1.535	0.000	-2.000
c3-c3-oh-ho	1	3.043	0.000	1.000
h1-c3-c3-oh	1	0.938	0.000	-3.000
h1-c3-c3-oh	1	0.346	180.000	-2.000
h1-c3-c3-oh	1	1.109	0.000	1.000
f-c3-c3-oh	1	0.825	180.000	-3.000
f-c3-c3-oh	1	1.170	0.000	-2.000
f-c3-c3-oh	1	3.564	180.000	1.000
oh-c3-c3-os	1	0.108	0.000	-3.000
oh-c3-c3-os	1	0.715	0.000	-2.000
oh-c3-c3-os	1	0.543	180.000	1.000
h1-c3-oh-ho	1	0.001	0.000	-3.000

h1-c3-oh-ho	1	1.079	0.000	-2.000
h1-c3-oh-ho	1	2.921	0.000	1.000
h2-c3-c3-oh	1	0.212	180.000	-3.000
h2-c3-c3-oh	1	0.553	0.000	-2.000
h2-c3-c3-oh	1	0.094	0.000	1.000
na-c3-c3-oh	1	0.080	180.000	-3.000
na-c3-c3-oh	1	0.398	180.000	-2.000
na-c3-c3-oh	1	0.152	0.000	1.000
oh-c3-c3-oh	1	0.983	180.000	-3.000
oh-c3-c3-oh	1	0.985	0.000	-2.000
oh-c3-c3-oh	1	3.435	180.000	1.000
f-c3-c3-hc	1	0.047	180.000	-3.000
f-c3-c3-hc	1	0.002	0.000	-2.000
f-c3-c3-hc	1	0.723	180.000	1.000
hc-c3-c3-oh	1	0.074	180.000	-3.000
hc-c3-c3-oh	1	0.086	0.000	-2.000
hc-c3-c3-oh	1	1.331	180.000	1.00

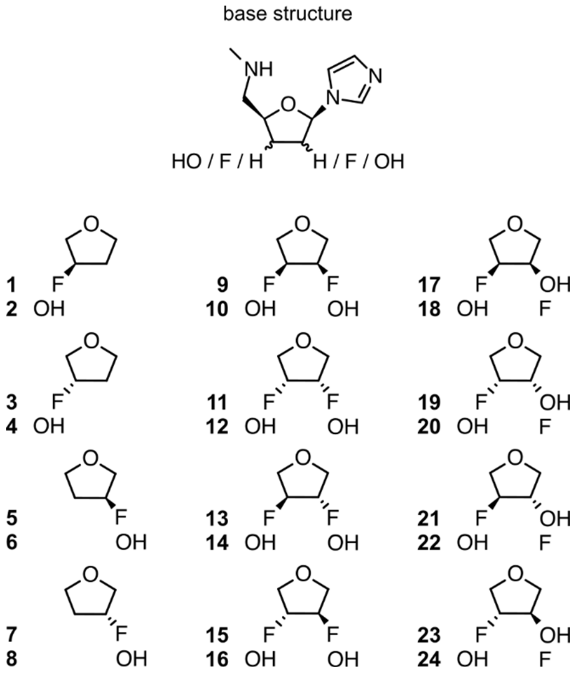

Figure S5. Set of 24 test furanose ring structures used to derive partial charges and parameterize torsional varaibles. Numbers in bold indicate the index number assigned to each molecule. The prepi files in **Tables S4–S27** correspond to each of the molecules **1–24**.

Table S4. prepi file for test molecule **1**.

```
    0    0    2

prepi file for test molecule 1
molecule.res
d01   INT  0
CORRECT     OMIT DU   BEG
  0.0000
   1  DUMM  DU    M    0  -1  -2     0.000      .0        .0      .00000
   2  DUMM  DU    M    1   0  -1     1.449      .0        .0      .00000
   3  DUMM  DU    M    2   1   0     1.523   111.21       .0      .00000
   4  C14   c3    M    3   2   1     1.540   111.208  -180.000 -0.059720
   5  H26   h1    E    4   3   2     1.090     0.000   -90.000  0.067110
   6  H27   h1    E    4   3   2     1.090   109.453  -120.008  0.069920
   7  H28   h1    E    4   3   2     1.090   109.453   120.008  0.068200
   8  N1    n3    M    4   3   2     1.460   109.490     0.000 -0.582000
   9  H15   hn    E    8   4   3     1.010   109.505   -59.943  0.307350
  10  C2    c3    M    8   4   3     1.460   109.490   180.000  0.021310
  11  H16   h1    E   10   8   4     1.090   109.453    59.992  0.066210
  12  H17   h1    E   10   8   4     1.090   109.453   -59.992  0.070510
  13  C3    c3    M   10   8   4     1.500   109.474  -180.000  0.145660
  14  O7    os    E   13  10   8     1.436   109.705    59.998 -0.414020
  15  H18   h1    E   13  10   8     1.092   107.677   -60.925  0.037450
  16  C4    c3    M   13  10   8     1.535   111.369   175.869  0.246920
  17  F13   f     E   16  13  10     1.337   107.684     3.503 -0.240910
  18  H19   h1    E   16  13  10     1.091   114.687   122.533  0.045800
  19  C5    c3    M   16  13  10     1.514   103.695  -111.946 -0.288320
  20  H20   hc    E   19  16  13     1.090   113.314  -151.801  0.095210
  21  H21   hc    E   19  16  13     1.091   110.440    87.060  0.117190
  22  C6    c3    M   19  16  13     1.522   102.231   -30.070  0.421640
  23  H22   h2    E   22  19  16     1.093   112.129   -75.233  0.028740
  24  N8    na    M   22  19  16     1.460   112.802   162.332 -0.080480
  25  C9    cc    M   24  22  19     1.370   127.533   -82.906 -0.279550
  26  H23   h4    E   25  24  22     1.084   122.222    -0.013  0.189140
  27  C10   cd    M   25  24  22     1.386   104.733   179.986  0.082870
  28  H24   h4    E   27  25  24     1.085   126.008  -179.997  0.114310
  29  N11   nd    M   27  25  24     1.325   111.679    -0.029 -0.529920
  30  C12   cc    M   29  27  25     1.313   105.418     0.040  0.178380
  31  H25   h5    E   30  29  27     1.085   127.386  -179.959  0.101000


LOOP
   C6   O7
  C12   N8

IMPROPER
   C6   C9   N8  C12
  C10  H23   C9   N8
   C9  H24  C10  N11
  H25   N8  C12  N11

DONE
STOP
```

Table S5. prepi file for test molecule **2**.

```
    0    0    2

prepi file for test molecule 2
molecule.res
d02   INT  0
CORRECT     OMIT DU   BEG
  0.0000
   1  DUMM  DU    M    0  -1  -2     0.000      .0        .0      .00000
   2  DUMM  DU    M    1   0  -1     1.449      .0        .0      .00000
   3  DUMM  DU    M    2   1   0     1.523   111.21       .0      .00000
   4  C14   c3    M    3   2   1     1.540   111.208  -180.000  0.029800
   5  H26   h1    E    4   3   2     1.090     0.000   -90.000  0.033120
   6  H27   h1    E    4   3   2     1.090   109.453  -120.008  0.042920
   7  H28   h1    E    4   3   2     1.090   109.453   120.008  0.051250
   8  N1    n3    M    4   3   2     1.460   109.490     0.000 -0.599120
   9  H15   hn    E    8   4   3     1.010   109.505   -59.943  0.327820
  10  C2    c3    M    8   4   3     1.460   109.490   180.000 -0.047330
  11  H16   h1    E   10   8   4     1.090   109.453    59.992  0.096770
  12  H17   h1    E   10   8   4     1.090   109.453   -59.992  0.101000
  13  C3    c3    M   10   8   4     1.500   109.474  -180.000  0.061960
  14  O7    os    E   13  10   8     1.436   109.705    59.998 -0.385660
  15  H18   h1    E   13  10   8     1.092   107.677   -60.925  0.082480
  16  C4    c3    M   13  10   8     1.535   111.369   175.869  0.152500
  17  O13   oh    S   16  13  10     1.410   107.655     3.504 -0.571350
  18  H29   ho    E   17  16  13     0.960   109.479   179.980  0.411670
  19  H19   h1    E   16  13  10     1.091   114.687   122.533  0.042430
  20  C5    c3    M   16  13  10     1.514   103.695  -111.946 -0.201770
  21  H20   hc    E   20  16  13     1.090   113.314  -151.801  0.088400
  22  H21   hc    E   20  16  13     1.091   110.440    87.060  0.082640
  23  C6    c3    M   20  16  13     1.522   102.231   -30.070  0.406100
  24  H22   h2    E   23  20  16     1.093   112.129   -75.233  0.015160
  25  N8    na    M   23  20  16     1.460   112.802   162.332 -0.105320
  26  C9    cc    M   25  23  20     1.370   127.533   -82.906 -0.261560
  27  H23   h4    E   26  25  23     1.084   122.222    -0.013  0.179170
  28  C10   cd    M   26  25  23     1.386   104.733   179.986  0.102290
  29  H24   h4    E   28  26  25     1.085   126.008  -179.997  0.108860
  30  N11   nd    M   28  26  25     1.325   111.679    -0.029 -0.551220
  31  C12   cc    M   30  28  26     1.313   105.418     0.040  0.210030
  32  H25   h5    E   31  30  28     1.085   127.386  -179.959  0.096960


LOOP
   C6   O7
  C12   N8

IMPROPER
   C6   C9   N8  C12
  C10  H23   C9   N8
   C9  H24  C10  N11
  H25   N8  C12  N11

DONE
STOP
```

Table S6. prepi file for test molecule **3**.

```
    0    0    2

prepi file for test molecule 3
molecule.res
d03   INT  0
CORRECT     OMIT DU   BEG
  0.0000
   1  DUMM  DU    M    0  -1  -2     0.000      .0        .0      .00000
   2  DUMM  DU    M    1   0  -1     1.449      .0        .0      .00000
   3  DUMM  DU    M    2   1   0     1.523   111.21       .0      .00000
   4  C14   c3    M    3   2   1     1.540   111.208  -180.000 -0.001620
   5  H26   h1    E    4   3   2     1.090     0.000   -90.000  0.053650
   6  H27   h1    E    4   3   2     1.090   109.453  -120.008  0.056200
   7  H28   h1    E    4   3   2     1.090   109.453   120.008  0.053020
   8  N1    n3    M    4   3   2     1.460   109.490     0.000 -0.584370
   9  H15   hn    E    8   4   3     1.010   109.505   -59.943  0.307680
  10  C2    c3    M    8   4   3     1.460   109.490   180.000 -0.038050
  11  H16   h1    E   10   8   4     1.090   109.453    59.992  0.064850
  12  H17   h1    E   10   8   4     1.090   109.453   -59.992  0.089220
  13  C3    c3    M   10   8   4     1.500   109.474  -180.000  0.190380
  14  O7    os    E   13  10   8     1.436   109.705    59.998 -0.422880
  15  H18   h1    E   13  10   8     1.092   107.677   -60.925  0.026740
  16  C4    c3    M   13  10   8     1.535   111.369   175.869  0.313400
  17  F13   f     E   16  13  10     1.336   109.044   133.530 -0.262980
  18  H19   h1    E   16  13  10     1.091   114.423    13.693  0.006730
  19  C5    c3    M   16  13  10     1.514   103.695  -111.946 -0.247740
  20  H20   hc    E   19  16  13     1.090   113.314  -151.801  0.089600
  21  H21   hc    E   19  16  13     1.091   110.440    87.060  0.083980
  22  C6    c3    M   19  16  13     1.522   102.231   -30.070  0.440730
  23  H22   h2    E   22  19  16     1.093   112.129   -75.233  0.020050
  24  N8    na    M   22  19  16     1.460   112.802   162.332 -0.083030
  25  C9    cc    M   24  22  19     1.370   127.533   -82.906 -0.262890
  26  H23   h4    E   25  24  22     1.084   122.222    -0.013  0.180270
  27  C10   cd    M   25  24  22     1.386   104.733   179.986  0.085770
  28  H24   h4    E   27  25  24     1.085   126.008  -179.997  0.112030
  29  N11   nd    M   27  25  24     1.325   111.679    -0.029 -0.514460
  30  C12   cc    M   29  27  25     1.313   105.418     0.040  0.129610
  31  H25   h5    E   30  29  27     1.085   127.386  -179.959  0.114110


LOOP
   C6   O7
  C12   N8

IMPROPER
   C6   C9   N8  C12
  C10  H23   C9   N8
   C9  H24  C10  N11
  H25   N8  C12  N11

DONE
STOP
```

Table S7. prepi file for test molecule **4**.

```
    0    0    2

prepi file for test molecule 4
molecule.res
d04   INT  0
CORRECT     OMIT DU   BEG
  0.0000
   1  DUMM  DU    M    0  -1  -2     0.000      .0        .0      .00000
   2  DUMM  DU    M    1   0  -1     1.449      .0        .0      .00000
   3  DUMM  DU    M    2   1   0     1.523   111.21       .0      .00000
   4  C14   c3    M    3   2   1     1.540   111.208  -180.000 -0.003800
   5  H26   h1    E    4   3   2     1.090     0.000   -90.000  0.051110
   6  H27   h1    E    4   3   2     1.090   109.453  -120.008  0.051230
   7  H28   h1    E    4   3   2     1.090   109.453   120.008  0.056200
   8  N1    n3    M    4   3   2     1.460   109.490     0.000 -0.597760
   9  H15   hn    E    8   4   3     1.010   109.505   -59.943  0.318570
  10  C2    c3    M    8   4   3     1.460   109.490   180.000  0.003160
  11  H16   h1    E   10   8   4     1.090   109.453    59.992  0.067250
  12  H17   h1    E   10   8   4     1.090   109.453   -59.992  0.076520
  13  C3    c3    M   10   8   4     1.500   109.474  -180.000  0.096560
  14  O7    os    E   13  10   8     1.436   109.705    59.998 -0.388990
  15  H18   h1    E   13  10   8     1.092   107.677   -60.925  0.060870
  16  C4    c3    M   13  10   8     1.535   111.369   175.869  0.276130
  17  O13   oh    S   16  13  10     1.410   109.046   133.545 -0.593130
  18  H29   ho    E   17  16  13     0.960   109.464  -179.983  0.384260
  19  H19   h1    E   16  13  10     1.091   114.423    13.693  0.029090
  20  C5    c3    M   16  13  10     1.514   103.695  -111.946 -0.293970
  21  H20   hc    E   20  16  13     1.090   113.314  -151.801  0.117380
  22  H21   hc    E   20  16  13     1.091   110.440    87.060  0.100010
  23  C6    c3    M   20  16  13     1.522   102.231   -30.070  0.377460
  24  H22   h2    E   23  20  16     1.093   112.129   -75.233  0.064320
  25  N8    na    M   23  20  16     1.460   112.802   162.332 -0.174620
  26  C9    cc    M   25  23  20     1.370   127.533   -82.906 -0.195560
  27  H23   h4    E   26  25  23     1.084   122.222    -0.013  0.168050
  28  C10   cd    M   26  25  23     1.386   104.733   179.986  0.046580
  29  H24   h4    E   28  26  25     1.085   126.008  -179.997  0.126610
  30  N11   nd    M   28  26  25     1.325   111.679    -0.029 -0.540060
  31  C12   cc    M   30  28  26     1.313   105.418     0.040  0.227060
  32  H25   h5    E   31  30  28     1.085   127.386  -179.959  0.089470


LOOP
   C6   O7
  C12   N8

IMPROPER
   C6   C9   N8  C12
  C10  H23   C9   N8
   C9  H24  C10  N11
  H25   N8  C12  N11

DONE
STOP
```

Table S8. prepi file for test molecule **5**.

```
    0    0    2

prepi file for test molecule 5
molecule.res
d05   INT  0
CORRECT     OMIT DU   BEG
  0.0000
   1  DUMM  DU    M    0  -1  -2     0.000      .0        .0      .00000
   2  DUMM  DU    M    1   0  -1     1.449      .0        .0      .00000
   3  DUMM  DU    M    2   1   0     1.523   111.21       .0      .00000
   4  C14   c3    M    3   2   1     1.540   111.208  -180.000  0.000690
   5  H26   h1    E    4   3   2     1.090     0.000   -90.000  0.047740
   6  H27   h1    E    4   3   2     1.090   109.453  -120.008  0.054090
   7  H28   h1    E    4   3   2     1.090   109.453   120.008  0.057240
   8  N1    n3    M    4   3   2     1.460   109.490     0.000 -0.559020
   9  H15   hn    E    8   4   3     1.010   109.505   -59.943  0.301920
  10  C2    c3    M    8   4   3     1.460   109.490   180.000 -0.120720
  11  H16   h1    E   10   8   4     1.090   109.453    59.992  0.094110
  12  H17   h1    E   10   8   4     1.090   109.453   -59.992  0.095470
  13  C3    c3    M   10   8   4     1.500   109.474  -180.000  0.296790
  14  O7    os    E   13  10   8     1.436   109.705    59.998 -0.398950
  15  H18   h1    E   13  10   8     1.092   107.677   -60.925  0.009810
  16  C4    c3    M   13  10   8     1.535   111.369   175.869 -0.223790
  17  H13   hc    E   16  13  10     1.089   109.031   133.558  0.086620
  18  H19   hc    E   16  13  10     1.091   114.423    13.693  0.089910
  19  C5    c3    M   16  13  10     1.514   103.695  -111.946  0.223760
  20  H20   h1    E   19  16  13     1.090   113.314  -151.801  0.039120
  21  F21   f     E   19  16  13     1.380   110.438    87.062 -0.228620
  22  C6    c3    M   19  16  13     1.522   102.231   -30.070  0.275220
  23  H22   h2    E   22  19  16     1.093   112.129   -75.233  0.045070
  24  N8    na    M   22  19  16     1.460   112.802   162.332 -0.006340
  25  C9    cc    M   24  22  19     1.370   127.533   -82.906 -0.306800
  26  H23   h4    E   25  24  22     1.084   122.222    -0.013  0.196860
  27  C10   cd    M   25  24  22     1.386   104.733   179.986  0.108590
  28  H24   h4    E   27  25  24     1.085   126.008  -179.997  0.105940
  29  N11   nd    M   27  25  24     1.325   111.679    -0.029 -0.514060
  30  C12   cc    M   29  27  25     1.313   105.418     0.040  0.104700
  31  H25   h5    E   30  29  27     1.085   127.386  -179.959  0.124650


LOOP
   C6   O7
  C12   N8

IMPROPER
   C6   C9   N8  C12
  C10  H23   C9   N8
   C9  H24  C10  N11
  H25   N8  C12  N11

DONE
STOP
```

Table S9. prepi file for test molecule **6**.

```
    0    0    2

prepi file for test molecule 6
molecule.res
d06   INT  0
CORRECT     OMIT DU   BEG
  0.0000
   1  DUMM  DU    M    0  -1  -2     0.000      .0        .0      .00000
   2  DUMM  DU    M    1   0  -1     1.449      .0        .0      .00000
   3  DUMM  DU    M    2   1   0     1.523   111.21       .0      .00000
   4  C14   c3    M    3   2   1     1.540   111.208  -180.000  0.082490
   5  H26   h1    E    4   3   2     1.090     0.000   -90.000  0.033300
   6  H27   h1    E    4   3   2     1.090   109.453  -120.008  0.029700
   7  H28   h1    E    4   3   2     1.090   109.453   120.008  0.026080
   8  N1    n3    M    4   3   2     1.460   109.490     0.000 -0.575440
   9  H15   hn    E    8   4   3     1.010   109.505   -59.943  0.292980
  10  C2    c3    M    8   4   3     1.460   109.490   180.000 -0.039400
  11  H16   h1    E   10   8   4     1.090   109.453    59.992  0.077120
  12  H17   h1    E   10   8   4     1.090   109.453   -59.992  0.068370
  13  C3    c3    M   10   8   4     1.500   109.474  -180.000  0.267620
  14  O7    os    E   13  10   8     1.436   109.705    59.998 -0.413300
  15  H18   h1    E   13  10   8     1.092   107.677   -60.925  0.022150
  16  C4    c3    M   13  10   8     1.535   111.369   175.869 -0.245860
  17  H13   hc    E   16  13  10     1.089   109.031   133.558  0.079630
  18  H19   hc    E   16  13  10     1.091   114.423    13.693  0.100490
  19  C5    c3    M   16  13  10     1.514   103.695  -111.946  0.222730
  20  H20   h1    E   19  16  13     1.090   113.314  -151.801  0.038140
  21  O21   oh    S   19  16  13     1.410   110.427    87.045 -0.556130
  22  H29   ho    E   21  19  16     0.961   109.504   179.965  0.370290
  23  C6    c3    M   19  16  13     1.522   102.231   -30.070  0.264220
  24  H22   h2    E   23  19  16     1.093   112.129   -75.233  0.029630
  25  N8    na    M   23  19  16     1.460   112.802   162.332  0.012620
  26  C9    cc    M   25  23  19     1.370   127.533   -82.906 -0.269770
  27  H23   h4    E   26  25  23     1.084   122.222    -0.013  0.173310
  28  C10   cd    M   26  25  23     1.386   104.733   179.986  0.098300
  29  H24   h4    E   28  26  25     1.085   126.008  -179.997  0.110690
  30  N11   nd    M   28  26  25     1.325   111.679    -0.029 -0.539750
  31  C12   cc    M   30  28  26     1.313   105.418     0.040  0.116840
  32  H25   h5    E   31  30  28     1.085   127.386  -179.959  0.122950


LOOP
   C6   O7
  C12   N8

IMPROPER
   C6    C9    N8  C12
  C10  H23    C9    N8
   C9  H24  C10  N11
  H25    N8  C12  N11

DONE
STOP
```

Table S10. prepi file for test molecule **7**.

```
    0    0    2

prepi file for test molecule 7
molecule.res
d07   INT  0
CORRECT     OMIT DU   BEG
  0.0000
   1  DUMM  DU    M    0  -1  -2     0.000      .0        .0      .00000
   2  DUMM  DU    M    1   0  -1     1.449      .0        .0      .00000
   3  DUMM  DU    M    2   1   0     1.523   111.21       .0      .00000
   4  C14   c3    M    3   2   1     1.540   111.208  -180.000 -0.023750
   5  H26   h1    E    4   3   2     1.090     0.000   -90.000  0.052560
   6  H27   h1    E    4   3   2     1.090   109.453  -120.008  0.061190
   7  H28   h1    E    4   3   2     1.090   109.453   120.008  0.056410
   8  N1    n3    M    4   3   2     1.460   109.490     0.000 -0.552590
   9  H15   hn    E    8   4   3     1.010   109.505   -59.943  0.296650
  10  C2    c3    M    8   4   3     1.460   109.490   180.000 -0.056710
  11  H16   h1    E   10   8   4     1.090   109.453    59.992  0.090270
  12  H17   h1    E   10   8   4     1.090   109.453   -59.992  0.081820
  13  C3    c3    M   10   8   4     1.500   109.474  -180.000  0.223060
  14  O7    os    E   13  10   8     1.436   109.705    59.998 -0.383380
  15  H18   h1    E   13  10   8     1.092   107.677   -60.925  0.044770
  16  C4    c3    M   13  10   8     1.535   111.369   175.869 -0.266160
  17  H13   hc    E   16  13  10     1.089   109.031   133.558  0.107300
  18  H19   hc    E   16  13  10     1.091   114.423    13.693  0.096120
  19  C5    c3    M   16  13  10     1.514   103.695  -111.946  0.302380
  20  F20   f     E   19  16  13     1.380   113.304  -151.791 -0.254260
  21  H21   h1    E   19  16  13     1.091   110.440    87.060  0.036800
  22  C6    c3    M   19  16  13     1.522   102.231   -30.070  0.155580
  23  H22   h2    E   22  19  16     1.093   112.129   -75.233  0.100040
  24  N8    na    M   22  19  16     1.460   112.802   162.332 -0.041620
  25  C9    cc    M   24  22  19     1.370   127.533   -82.906 -0.248990
  26  H23   h4    E   25  24  22     1.084   122.222    -0.013  0.182460
  27  C10   cd    M   25  24  22     1.386   104.733   179.986  0.078180
  28  H24   h4    E   27  25  24     1.085   126.008  -179.997  0.115390
  29  N11   nd    M   27  25  24     1.325   111.679    -0.029 -0.538490
  30  C12   cc    M   29  27  25     1.313   105.418     0.040  0.180720
  31  H25   h5    E   30  29  27     1.085   127.386  -179.959  0.104250


LOOP
   C6   O7
  C12   N8

IMPROPER
   C6   C9   N8  C12
  C10  H23   C9   N8
   C9  H24  C10  N11
  H25   N8  C12  N11

DONE
STOP
```

Table S11. prepi file for test molecule **8**.

```
    0    0    2

prepi file for test molecule 8
molecule.res
d08   INT  0
CORRECT     OMIT DU   BEG
  0.0000
   1  DUMM  DU    M    0  -1  -2     0.000      .0        .0      .00000
   2  DUMM  DU    M    1   0  -1     1.449      .0        .0      .00000
   3  DUMM  DU    M    2   1   0     1.523   111.21       .0      .00000
   4  C14   c3    M    3   2   1     1.540   111.208  -180.000 -0.105950
   5  H26   h1    E    4   3   2     1.090     0.000   -90.000  0.073600
   6  H27   h1    E    4   3   2     1.090   109.453  -120.008  0.075820
   7  H28   h1    E    4   3   2     1.090   109.453   120.008  0.083530
   8  N1    n3    M    4   3   2     1.460   109.490     0.000 -0.526810
   9  H15   hn    E    8   4   3     1.010   109.505   -59.943  0.298490
  10  C2    c3    M    8   4   3     1.460   109.490   180.000 -0.100730
  11  H16   h1    E   10   8   4     1.090   109.453    59.992  0.079520
  12  H17   h1    E   10   8   4     1.090   109.453   -59.992  0.100770
  13  C3    c3    M   10   8   4     1.500   109.474  -180.000  0.243640
  14  O7    os    E   13  10   8     1.436   109.705    59.998 -0.381000
  15  H18   h1    E   13  10   8     1.092   107.677   -60.925  0.022250
  16  C4    c3    M   13  10   8     1.535   111.369   175.869 -0.022490
  17  H13   hc    E   16  13  10     1.089   109.031   133.558  0.038810
  18  H19   hc    E   16  13  10     1.091   114.423    13.693  0.021700
  19  C5    c3    M   16  13  10     1.514   103.695  -111.946  0.098240
  20  O20   oh    S   19  16  13     1.410   113.305  -151.801 -0.533350
  21  H29   ho    E   20  19  16     0.961   109.496   179.992  0.370660
  22  H21   h1    E   19  16  13     1.091   110.440    87.060  0.074320
  23  C6    c3    M   19  16  13     1.522   102.231   -30.070  0.120960
  24  H22   h2    E   23  19  16     1.093   112.129   -75.233  0.101230
  25  N8    na    M   23  19  16     1.460   112.802   162.332  0.083640
  26  C9    cc    M   25  23  19     1.370   127.533   -82.906 -0.294680
  27  H23   h4    E   26  25  23     1.084   122.222    -0.013  0.183740
  28  C10   cd    M   26  25  23     1.386   104.733   179.986  0.052460
  29  H24   h4    E   28  26  25     1.085   126.008  -179.997  0.130680
  30  N11   nd    M   28  26  25     1.325   111.679    -0.029 -0.504900
  31  C12   cc    M   30  28  26     1.313   105.418     0.040  0.093350
  32  H25   h5    E   31  30  28     1.085   127.386  -179.959  0.122500


LOOP
   C6   O7
  C12   N8

IMPROPER
   C6    C9    N8   C12
  C10   H23    C9    N8
   C9   H24   C10   N11
  H25    N8   C12   N11

DONE
STOP
```

Table S12. prepi file for test molecule **9**.

```
    0    0    2

prepi file for test molecule 9
molecule.res
d09   INT  0
CORRECT     OMIT DU   BEG
  0.0000
   1  DUMM  DU    M    0  -1  -2     0.000      .0        .0      .00000
   2  DUMM  DU    M    1   0  -1     1.449      .0        .0      .00000
   3  DUMM  DU    M    2   1   0     1.523   111.21       .0      .00000
   4  C14   c3    M    3   2   1     1.540   111.208  -180.000 -0.059940
   5  H26   h1    E    4   3   2     1.090     0.000   -90.000  0.066500
   6  H27   h1    E    4   3   2     1.090   109.453  -120.008  0.062010
   7  H28   h1    E    4   3   2     1.090   109.453   120.008  0.072840
   8  N1    n3    M    4   3   2     1.460   109.490     0.000 -0.569960
   9  H15   hn    E    8   4   3     1.010   109.505   -59.943  0.296280
  10  C2    c3    M    8   4   3     1.460   109.490   180.000  0.075380
  11  H16   h1    E   10   8   4     1.090   109.453    59.992  0.053950
  12  H17   h1    E   10   8   4     1.090   109.453   -59.992  0.051040
  13  C3    c3    M   10   8   4     1.500   109.474  -180.000  0.135060
  14  O7    os    E   13  10   8     1.436   109.705    59.998 -0.387240
  15  H18   h1    E   13  10   8     1.092   107.677   -60.925  0.024840
  16  C4    c3    M   13  10   8     1.535   111.369   175.869  0.189810
  17  H13   h1    E   16  13  10     1.089   109.031   133.558  0.041300
  18  F19   f     E   16  13  10     1.380   114.456    13.685 -0.220420
  19  C5    c3    M   16  13  10     1.514   103.695  -111.946  0.204240
  20  H20   h1    E   19  16  13     1.090   113.314  -151.801  0.039070
  21  F21   f     E   19  16  13     1.380   110.438    87.062 -0.223530
  22  C6    c3    M   19  16  13     1.522   102.231   -30.070  0.280140
  23  H22   h2    E   22  19  16     1.093   112.129   -75.233  0.036700
  24  N8    na    M   22  19  16     1.460   112.802   162.332  0.036270
  25  C9    cc    M   24  22  19     1.370   127.533   -82.906 -0.330430
  26  H23   h4    E   25  24  22     1.084   122.222    -0.013  0.210320
  27  C10   cd    M   25  24  22     1.386   104.733   179.986  0.072230
  28  H24   h4    E   27  25  24     1.085   126.008  -179.997  0.124510
  29  N11   nd    M   27  25  24     1.325   111.679    -0.029 -0.509160
  30  C12   cc    M   29  27  25     1.313   105.418     0.040  0.119520
  31  H25   h5    E   30  29  27     1.085   127.386  -179.959  0.108670


LOOP
   C6   O7
  C12   N8

IMPROPER
   C6   C9   N8  C12
  C10  H23   C9   N8
   C9  H24  C10  N11
  H25   N8  C12  N11

DONE
STOP
```

Table S13. prepi file for test molecule **10**.

```
    0    0    2

prepi file for test molecule 10
molecule.res
d10   INT  0
CORRECT     OMIT DU   BEG
  0.0000
   1  DUMM  DU    M    0  -1  -2     0.000      .0        .0      .00000
   2  DUMM  DU    M    1   0  -1     1.449      .0        .0      .00000
   3  DUMM  DU    M    2   1   0     1.523   111.21       .0      .00000
   4  C14   c3    M    3   2   1     1.540   111.208  -180.000  0.025610
   5  H26   h1    E    4   3   2     1.090     0.000   -90.000  0.047200
   6  H27   h1    E    4   3   2     1.090   109.453  -120.008  0.050770
   7  H28   h1    E    4   3   2     1.090   109.453   120.008  0.054110
   8  N1    n3    M    4   3   2     1.460   109.490     0.000 -0.633110
   9  H15   hn    E    8   4   3     1.010   109.505   -59.943  0.314600
  10  C2    c3    M    8   4   3     1.460   109.490   180.000 -0.021980
  11  H16   h1    E   10   8   4     1.090   109.453    59.992  0.075500
  12  H17   h1    E   10   8   4     1.090   109.453   -59.992  0.072890
  13  C3    c3    M   10   8   4     1.500   109.474  -180.000  0.315180
  14  O7    os    E   13  10   8     1.436   109.705    59.998 -0.414600
  15  H18   h1    E   13  10   8     1.092   107.677   -60.925 -0.007110
  16  C4    c3    M   13  10   8     1.535   111.369   175.869  0.194420
  17  H13   h1    E   16  13  10     1.089   109.031   133.558  0.043660
  18  O19   oh    S   16  13  10     1.409   114.435    13.689 -0.607030
  19  H29   ho    E   18  16  13     0.960   109.498  -179.972  0.429550
  20  C5    c3    M   16  13  10     1.514   103.695  -111.946 -0.144230
  21  H20   h1    E   20  16  13     1.090   113.314  -151.801  0.143910
  22  O21   oh    S   20  16  13     1.410   110.427    87.045 -0.512400
  23  H30   ho    E   22  20  16     0.961   109.504   179.965  0.403240
  24  C6    c3    M   20  16  13     1.522   102.231   -30.070  0.207080
  25  H22   h2    E   24  20  16     1.093   112.129   -75.233  0.071380
  26  N8    na    M   24  20  16     1.460   112.802   162.332  0.150680
  27  C9    cc    M   26  24  20     1.370   127.533   -82.906 -0.417430
  28  H23   h4    E   27  26  24     1.084   122.222    -0.013  0.221360
  29  C10   cd    M   27  26  24     1.386   104.733   179.986  0.154080
  30  H24   h4    E   29  27  26     1.085   126.008  -179.997  0.106540
  31  N11   nd    M   29  27  26     1.325   111.679    -0.029 -0.528210
  32  C12   cc    M   31  29  27     1.313   105.418     0.040  0.056480
  33  H25   h5    E   32  31  29     1.085   127.386  -179.959  0.147860


LOOP
   C6   O7
  C12   N8

IMPROPER
   C6   C9   N8  C12
  C10  H23   C9   N8
   C9  H24  C10  N11
  H25   N8  C12  N11

DONE
STOP
```

Table S14. prepi file for test molecule **11**.

```
    0    0    2

prepi file for test molecule 11
molecule.res
d11   INT  0
CORRECT     OMIT DU   BEG
  0.0000
   1  DUMM  DU    M    0  -1  -2     0.000      .0        .0      .00000
   2  DUMM  DU    M    1   0  -1     1.449      .0        .0      .00000
   3  DUMM  DU    M    2   1   0     1.523   111.21       .0      .00000
   4  C14   c3    M    3   2   1     1.540   111.208  -180.000 -0.151860
   5  H26   h1    E    4   3   2     1.090     0.000   -90.000  0.085290
   6  H27   h1    E    4   3   2     1.090   109.453  -120.008  0.090040
   7  H28   h1    E    4   3   2     1.090   109.453   120.008  0.091780
   8  N1    n3    M    4   3   2     1.460   109.490     0.000 -0.561760
   9  H15   hn    E    8   4   3     1.010   109.505   -59.943  0.308260
  10  C2    c3    M    8   4   3     1.460   109.490   180.000  0.138900
  11  H16   h1    E   10   8   4     1.090   109.453    59.992  0.028320
  12  H17   h1    E   10   8   4     1.090   109.453   -59.992  0.046620
  13  C3    c3    M   10   8   4     1.500   109.474  -180.000  0.078310
  14  O7    os    E   13  10   8     1.436   109.705    59.998 -0.402780
  15  H18   h1    E   13  10   8     1.092   107.677   -60.925  0.052390
  16  C4    c3    M   13  10   8     1.535   111.369   175.869  0.252330
  17  F13   f     E   16  13  10     1.379   109.041   133.545 -0.230430
  18  H19   h1    E   16  13  10     1.091   114.423    13.693  0.021510
  19  C5    c3    M   16  13  10     1.514   103.695  -111.946  0.116160
  20  F20   f     E   19  16  13     1.380   113.304  -151.791 -0.225680
  21  H21   h1    E   19  16  13     1.091   110.440    87.060  0.069560
  22  C6    c3    M   19  16  13     1.522   102.231   -30.070  0.350840
  23  H22   h2    E   22  19  16     1.093   112.129   -75.233  0.056810
  24  N8    na    M   22  19  16     1.460   112.802   162.332 -0.084750
  25  C9    cc    M   24  22  19     1.370   127.533   -82.906 -0.243200
  26  H23   h4    E   25  24  22     1.084   122.222    -0.013  0.178340
  27  C10   cd    M   25  24  22     1.386   104.733   179.986  0.068060
  28  H24   h4    E   27  25  24     1.085   126.008  -179.997  0.125340
  29  N11   nd    M   27  25  24     1.325   111.679    -0.029 -0.522610
  30  C12   cc    M   29  27  25     1.313   105.418     0.040  0.151480
  31  H25   h5    E   30  29  27     1.085   127.386  -179.959  0.112730


LOOP
   C6   O7
  C12   N8

IMPROPER
   C6   C9   N8  C12
  C10  H23   C9   N8
   C9  H24  C10  N11
  H25   N8  C12  N11

DONE
STOP
```

Table S15. prepi file for test molecule **12**.

```
    0    0    2

prepi file for test molecule 12
molecule.res
d12   INT  0
CORRECT     OMIT DU   BEG
  0.0000
   1  DUMM  DU    M    0  -1  -2     0.000      .0        .0      .00000
   2  DUMM  DU    M    1   0  -1     1.449      .0        .0      .00000
   3  DUMM  DU    M    2   1   0     1.523   111.21       .0      .00000
   4  C14   c3    M    3   2   1     1.540   111.208  -180.000  0.066610
   5  H26   h1    E    4   3   2     1.090     0.000   -90.000  0.028030
   6  H27   h1    E    4   3   2     1.090   109.453  -120.008  0.035940
   7  H28   h1    E    4   3   2     1.090   109.453   120.008  0.036770
   8  N1    n3    M    4   3   2     1.460   109.490     0.000 -0.626490
   9  H15   hn    E    8   4   3     1.010   109.505   -59.943  0.310220
  10  C2    c3    M    8   4   3     1.460   109.490   180.000  0.112810
  11  H16   h1    E   10   8   4     1.090   109.453    59.992  0.029630
  12  H17   h1    E   10   8   4     1.090   109.453   -59.992  0.041470
  13  C3    c3    M   10   8   4     1.500   109.474  -180.000  0.136600
  14  O7    os    E   13  10   8     1.436   109.705    59.998 -0.384260
  15  H18   h1    E   13  10   8     1.092   107.677   -60.925  0.050900
  16  C4    c3    M   13  10   8     1.535   111.369   175.869  0.200380
  17  O13   oh    S   16  13  10     1.410   109.046   133.545 -0.578810
  18  H29   ho    E   17  16  13     0.960   109.464  -179.983  0.387920
  19  H19   h1    E   16  13  10     1.091   114.423    13.693  0.020720
  20  C5    c3    M   16  13  10     1.514   103.695  -111.946  0.242270
  21  O20   oh    S   20  16  13     1.410   113.305  -151.801 -0.589780
  22  H30   ho    E   21  20  16     0.961   109.496   179.992  0.398720
  23  H21   h1    E   20  16  13     1.091   110.440    87.060  0.006540
  24  C6    c3    M   20  16  13     1.522   102.231   -30.070  0.129050
  25  H22   h2    E   24  20  16     1.093   112.129   -75.233  0.107570
  26  N8    na    M   24  20  16     1.460   112.802   162.332 -0.005390
  27  C9    cc    M   26  24  20     1.370   127.533   -82.906 -0.255030
  28  H23   h4    E   27  26  24     1.084   122.222    -0.013  0.179380
  29  C10   cd    M   27  26  24     1.386   104.733   179.986  0.050800
  30  H24   h4    E   29  27  26     1.085   126.008  -179.997  0.124870
  31  N11   nd    M   29  27  26     1.325   111.679    -0.029 -0.524770
  32  C12   cc    M   31  29  27     1.313   105.418     0.040  0.169500
  33  H25   h5    E   32  31  29     1.085   127.386  -179.959  0.097830


LOOP
   C6   O7
  C12   N8

IMPROPER
   C6   C9   N8  C12
  C10  H23   C9   N8
   C9  H24  C10  N11
  H25   N8  C12  N11

DONE
STOP
```

Table S16. prepi file for test molecule **13**.

```
    0    0    2

prepi file for test molecule 13
molecule.res
d13   INT  0
CORRECT     OMIT DU   BEG
  0.0000
   1  DUMM  DU    M    0  -1  -2     0.000      .0        .0      .00000
   2  DUMM  DU    M    1   0  -1     1.449      .0        .0      .00000
   3  DUMM  DU    M    2   1   0     1.523   111.21       .0      .00000
   4  C14   c3    M    3   2   1     1.540   111.208  -180.000  0.026440
   5  H26   h1    E    4   3   2     1.090     0.000   -90.000  0.046790
   6  H27   h1    E    4   3   2     1.090   109.453  -120.008  0.038420
   7  H28   h1    E    4   3   2     1.090   109.453   120.008  0.049730
   8  N1    n3    M    4   3   2     1.460   109.490     0.000 -0.585900
   9  H15   hn    E    8   4   3     1.010   109.505   -59.943  0.309580
  10  C2    c3    M    8   4   3     1.460   109.490   180.000 -0.008460
  11  H16   h1    E   10   8   4     1.090   109.453    59.992  0.080910
  12  H17   h1    E   10   8   4     1.090   109.453   -59.992  0.086320
  13  C3    c3    M   10   8   4     1.500   109.474  -180.000  0.083850
  14  O7    os    E   13  10   8     1.436   109.705    59.998 -0.380950
  15  H18   h1    E   13  10   8     1.092   107.677   -60.925  0.064310
  16  C4    c3    M   13  10   8     1.535   111.369   175.869  0.209630
  17  H13   h1    E   16  13  10     1.089   109.031   133.558  0.057350
  18  F19   f     E   16  13  10     1.380   114.456    13.685 -0.233750
  19  C5    c3    M   16  13  10     1.514   103.695  -111.946  0.128850
  20  F20   f     E   19  16  13     1.380   113.304  -151.791 -0.235080
  21  H21   h1    E   19  16  13     1.091   110.440    87.060  0.084210
  22  C6    c3    M   19  16  13     1.522   102.231   -30.070  0.330850
  23  H22   h2    E   22  19  16     1.093   112.129   -75.233  0.061070
  24  N8    na    M   22  19  16     1.460   112.802   162.332 -0.097220
  25  C9    cc    M   24  22  19     1.370   127.533   -82.906 -0.269370
  26  H23   h4    E   25  24  22     1.084   122.222    -0.013  0.185470
  27  C10   cd    M   25  24  22     1.386   104.733   179.986  0.100310
  28  H24   h4    E   27  25  24     1.085   126.008  -179.997  0.113520
  29  N11   nd    M   27  25  24     1.325   111.679    -0.029 -0.527990
  30  C12   cc    M   29  27  25     1.313   105.418     0.040  0.182080
  31  H25   h5    E   30  29  27     1.085   127.386  -179.959  0.099030


LOOP
   C6   O7
  C12   N8

IMPROPER
   C6   C9   N8  C12
  C10  H23   C9   N8
   C9  H24  C10  N11
  H25   N8  C12  N11

DONE
STOP
```

Table S17. prepi file for test molecule **14**.

```
    0    0    2

prepi file for test molecule 14
molecule.res
d14   INT  0
CORRECT     OMIT DU   BEG
  0.0000
   1  DUMM  DU    M    0  -1  -2     0.000      .0        .0      .00000
   2  DUMM  DU    M    1   0  -1     1.449      .0        .0      .00000
   3  DUMM  DU    M    2   1   0     1.523   111.21       .0      .00000
   4  C14   c3    M    3   2   1     1.540   111.208  -180.000  0.059170
   5  H26   h1    E    4   3   2     1.090     0.000   -90.000  0.040410
   6  H27   h1    E    4   3   2     1.090   109.453  -120.008  0.033560
   7  H28   h1    E    4   3   2     1.090   109.453   120.008  0.051290
   8  N1    n3    M    4   3   2     1.460   109.490     0.000 -0.661440
   9  H15   hn    E    8   4   3     1.010   109.505   -59.943  0.339740
  10  C2    c3    M    8   4   3     1.460   109.490   180.000  0.023680
  11  H16   h1    E   10   8   4     1.090   109.453    59.992  0.088790
  12  H17   h1    E   10   8   4     1.090   109.453   -59.992  0.068110
  13  C3    c3    M   10   8   4     1.500   109.474  -180.000  0.034180
  14  O7    os    E   13  10   8     1.436   109.705    59.998 -0.394840
  15  H18   h1    E   13  10   8     1.092   107.677   -60.925  0.080940
  16  C4    c3    M   13  10   8     1.535   111.369   175.869  0.281900
  17  H13   h1    E   16  13  10     1.089   109.031   133.558  0.027500
  18  O19   oh    S   16  13  10     1.409   114.435    13.689 -0.610830
  19  H29   ho    E   18  16  13     0.960   109.498  -179.972  0.409480
  20  C5    c3    M   16  13  10     1.514   103.695  -111.946 -0.002500
  21  O20   oh    S   20  16  13     1.410   113.305  -151.801 -0.549490
  22  H30   ho    E   21  20  16     0.961   109.496   179.992  0.386420
  23  H21   h1    E   20  16  13     1.091   110.440    87.060  0.086390
  24  C6    c3    M   20  16  13     1.522   102.231   -30.070  0.379370
  25  H22   h2    E   24  20  16     1.093   112.129   -75.233  0.036810
  26  N8    na    M   24  20  16     1.460   112.802   162.332 -0.050190
  27  C9    cc    M   26  24  20     1.370   127.533   -82.906 -0.230000
  28  H23   h4    E   27  26  24     1.084   122.222    -0.013  0.159560
  29  C10   cd    M   27  26  24     1.386   104.733   179.986  0.056930
  30  H24   h4    E   29  27  26     1.085   126.008  -179.997  0.123410
  31  N11   nd    M   29  27  26     1.325   111.679    -0.029 -0.504360
  32  C12   cc    M   31  29  27     1.313   105.418     0.040  0.114200
  33  H25   h5    E   32  31  29     1.085   127.386  -179.959  0.121810


LOOP
   C6   O7
  C12   N8

IMPROPER
   C6   C9   N8  C12
  C10  H23   C9   N8
   C9  H24  C10  N11
  H25   N8  C12  N11

DONE
STOP
```

Table S18. prepi file for test molecule **15**.

```
    0    0    2

prepi file for test molecule 15
molecule.res
d15   INT  0
CORRECT     OMIT DU   BEG
  0.0000
   1  DUMM  DU    M    0  -1  -2     0.000      .0        .0      .00000
   2  DUMM  DU    M    1   0  -1     1.449      .0        .0      .00000
   3  DUMM  DU    M    2   1   0     1.523   111.21       .0      .00000
   4  C14   c3    M    3   2   1     1.540   111.208  -180.000 -0.027640
   5  H26   h1    E    4   3   2     1.090     0.000   -90.000  0.060770
   6  H27   h1    E    4   3   2     1.090   109.453  -120.008  0.060060
   7  H28   h1    E    4   3   2     1.090   109.453   120.008  0.062480
   8  N1    n3    M    4   3   2     1.460   109.490     0.000 -0.572670
   9  H15   hn    E    8   4   3     1.010   109.505   -59.943  0.305260
  10  C2    c3    M    8   4   3     1.460   109.490   180.000 -0.005130
  11  H16   h1    E   10   8   4     1.090   109.453    59.992  0.080100
  12  H17   h1    E   10   8   4     1.090   109.453   -59.992  0.072340
  13  C3    c3    M   10   8   4     1.500   109.474  -180.000  0.112140
  14  O7    os    E   13  10   8     1.436   109.705    59.998 -0.410480
  15  H18   h1    E   13  10   8     1.092   107.677   -60.925  0.051440
  16  C4    c3    M   13  10   8     1.535   111.369   175.869  0.222050
  17  F13   f     E   16  13  10     1.379   109.041   133.545 -0.230590
  18  H19   h1    E   16  13  10     1.091   114.423    13.693  0.036780
  19  C5    c3    M   16  13  10     1.514   103.695  -111.946  0.126170
  20  H20   h1    E   19  16  13     1.090   113.314  -151.801  0.053230
  21  F21   f     E   19  16  13     1.380   110.438    87.062 -0.213210
  22  C6    c3    M   19  16  13     1.522   102.231   -30.070  0.465440
  23  H22   h2    E   22  19  16     1.093   112.129   -75.233  0.012420
  24  N8    na    M   22  19  16     1.460   112.802   162.332 -0.170820
  25  C9    cc    M   24  22  19     1.370   127.533   -82.906 -0.242700
  26  H23   h4    E   25  24  22     1.084   122.222    -0.013  0.180370
  27  C10   cd    M   25  24  22     1.386   104.733   179.986  0.073380
  28  H24   h4    E   27  25  24     1.085   126.008  -179.997  0.122380
  29  N11   nd    M   27  25  24     1.325   111.679    -0.029 -0.532340
  30  C12   cc    M   29  27  25     1.313   105.418     0.040  0.212030
  31  H25   h5    E   30  29  27     1.085   127.386  -179.959  0.096740


LOOP
   C6   O7
  C12   N8

IMPROPER
   C6   C9   N8  C12
  C10  H23   C9   N8
   C9  H24  C10  N11
  H25   N8  C12  N11

DONE
STOP
```

Table S19. prepi file for test molecule **16**.

```
    0    0    2

prepi file for test molecule 16
molecule.res
d16   INT  0
CORRECT     OMIT DU   BEG
  0.0000
   1  DUMM  DU    M    0  -1  -2     0.000      .0        .0      .00000
   2  DUMM  DU    M    1   0  -1     1.449      .0        .0      .00000
   3  DUMM  DU    M    2   1   0     1.523   111.21       .0      .00000
   4  C14   c3    M    3   2   1     1.540   111.208  -180.000 -0.069830
   5  H26   h1    E    4   3   2     1.090     0.000   -90.000  0.062290
   6  H27   h1    E    4   3   2     1.090   109.453  -120.008  0.066880
   7  H28   h1    E    4   3   2     1.090   109.453   120.008  0.077290
   8  N1    n3    M    4   3   2     1.460   109.490     0.000 -0.555310
   9  H15   hn    E    8   4   3     1.010   109.505   -59.943  0.302370
  10  C2    c3    M    8   4   3     1.460   109.490   180.000  0.041570
  11  H16   h1    E   10   8   4     1.090   109.453    59.992  0.058590
  12  H17   h1    E   10   8   4     1.090   109.453   -59.992  0.068600
  13  C3    c3    M   10   8   4     1.500   109.474  -180.000  0.037220
  14  O7    os    E   13  10   8     1.436   109.705    59.998 -0.349400
  15  H18   h1    E   13  10   8     1.092   107.677   -60.925  0.076470
  16  C4    c3    M   13  10   8     1.535   111.369   175.869  0.263810
  17  O13   oh    S   16  13  10     1.410   109.046   133.545 -0.578150
  18  H29   ho    E   17  16  13     0.960   109.464  -179.983  0.377640
  19  H19   h1    E   16  13  10     1.091   114.423    13.693  0.035720
  20  C5    c3    M   16  13  10     1.514   103.695  -111.946 -0.018440
  21  H20   h1    E   20  16  13     1.090   113.314  -151.801  0.096860
  22  O21   oh    S   20  16  13     1.410   110.427    87.045 -0.540050
  23  H30   ho    E   22  20  16     0.961   109.504   179.965  0.410840
  24  C6    c3    M   20  16  13     1.522   102.231   -30.070  0.290690
  25  H22   h2    E   24  20  16     1.093   112.129   -75.233  0.069080
  26  N8    na    M   24  20  16     1.460   112.802   162.332 -0.129630
  27  C9    cc    M   26  24  20     1.370   127.533   -82.906 -0.239930
  28  H23   h4    E   27  26  24     1.084   122.222    -0.013  0.179280
  29  C10   cd    M   27  26  24     1.386   104.733   179.986  0.088250
  30  H24   h4    E   29  27  26     1.085   126.008  -179.997  0.115540
  31  N11   nd    M   29  27  26     1.325   111.679    -0.029 -0.541120
  32  C12   cc    M   31  29  27     1.313   105.418     0.040  0.201080
  33  H25   h5    E   32  31  29     1.085   127.386  -179.959  0.101790


LOOP
   C6   O7
  C12   N8

IMPROPER
   C6   C9   N8  C12
  C10  H23   C9   N8
   C9  H24  C10  N11
  H25   N8  C12  N11

DONE
STOP
```

Table S20. prepi file for test molecule **17**.

```
    0    0    2

prepi file for test molecule 17
molecule.res
d17   INT  0
CORRECT     OMIT DU   BEG
  0.0000
   1  DUMM  DU    M    0  -1  -2     0.000      .0        .0      .00000
   2  DUMM  DU    M    1   0  -1     1.449      .0        .0      .00000
   3  DUMM  DU    M    2   1   0     1.523   111.21       .0      .00000
   4  C14   c3    M    3   2   1     1.540   111.208  -180.000  0.032290
   5  H26   h1    E    4   3   2     1.090     0.000   -90.000  0.042730
   6  H27   h1    E    4   3   2     1.090   109.453  -120.008  0.045920
   7  H28   h1    E    4   3   2     1.090   109.453   120.008  0.050050
   8  N1    n3    M    4   3   2     1.460   109.490     0.000 -0.626970
   9  H15   hn    E    8   4   3     1.010   109.505   -59.943  0.319150
  10  C2    c3    M    8   4   3     1.460   109.490   180.000  0.030070
  11  H16   h1    E   10   8   4     1.090   109.453    59.992  0.065840
  12  H17   h1    E   10   8   4     1.090   109.453   -59.992  0.079790
  13  C3    c3    M   10   8   4     1.500   109.474  -180.000  0.078150
  14  O7    os    E   13  10   8     1.436   109.705    59.998 -0.376390
  15  H18   h1    E   13  10   8     1.092   107.677   -60.925  0.057880
  16  C4    c3    M   13  10   8     1.535   111.369   175.869  0.308170
  17  H13   h1    E   16  13  10     1.089   109.031   133.558  0.000790
  18  F19   f     E   16  13  10     1.380   114.456    13.685 -0.243150
  19  C5    c3    M   16  13  10     1.514   103.695  -111.946  0.107400
  20  H20   h1    E   19  16  13     1.090   113.314  -151.801  0.042750
  21  O21   oh    S   19  16  13     1.410   110.427    87.045 -0.554470
  22  H29   ho    E   21  19  16     0.961   109.504   179.965  0.397910
  23  C6    c3    M   19  16  13     1.522   102.231   -30.070  0.267330
  24  H22   h2    E   23  19  16     1.093   112.129   -75.233  0.040080
  25  N8    na    M   23  19  16     1.460   112.802   162.332  0.029250
  26  C9    cc    M   25  23  19     1.370   127.533   -82.906 -0.365280
  27  H23   h4    E   26  25  23     1.084   122.222    -0.013  0.213180
  28  C10   cd    M   26  25  23     1.386   104.733   179.986  0.139880
  29  H24   h4    E   28  26  25     1.085   126.008  -179.997  0.109830
  30  N11   nd    M   28  26  25     1.325   111.679    -0.029 -0.551270
  31  C12   cc    M   30  28  26     1.313   105.418     0.040  0.141750
  32  H25   h5    E   31  30  28     1.085   127.386  -179.959  0.117340


LOOP
   C6   O7
  C12   N8

IMPROPER
   C6   C9   N8  C12
  C10  H23   C9   N8
   C9  H24  C10  N11
  H25   N8  C12  N11

DONE
STOP
```

Table S21. prepi file for test molecule **18**.

```
    0    0    2

prepi file for test molecule 18
molecule.res
d18   INT  0
CORRECT     OMIT DU   BEG
  0.0000
   1  DUMM  DU    M    0  -1  -2     0.000      .0        .0      .00000
   2  DUMM  DU    M    1   0  -1     1.449      .0        .0      .00000
   3  DUMM  DU    M    2   1   0     1.523   111.21       .0      .00000
   4  C14   c3    M    3   2   1     1.540   111.208  -180.000 -0.041080
   5  H26   h1    E    4   3   2     1.090     0.000   -90.000  0.067200
   6  H27   h1    E    4   3   2     1.090   109.453  -120.008  0.056440
   7  H28   h1    E    4   3   2     1.090   109.453   120.008  0.070860
   8  N1    n3    M    4   3   2     1.460   109.490     0.000 -0.598610
   9  H15   hn    E    8   4   3     1.010   109.505   -59.943  0.318860
  10  C2    c3    M    8   4   3     1.460   109.490   180.000  0.002610
  11  H16   h1    E   10   8   4     1.090   109.453    59.992  0.072740
  12  H17   h1    E   10   8   4     1.090   109.453   -59.992  0.072210
  13  C3    c3    M   10   8   4     1.500   109.474  -180.000  0.252750
  14  O7    os    E   13  10   8     1.436   109.705    59.998 -0.442600
  15  H18   h1    E   13  10   8     1.092   107.677   -60.925 -0.007980
  16  C4    c3    M   13  10   8     1.535   111.369   175.869  0.121320
  17  H13   h1    E   16  13  10     1.089   109.031   133.558  0.038840
  18  O19   oh    S   16  13  10     1.409   114.435    13.689 -0.602330
  19  H29   ho    E   18  16  13     0.960   109.498  -179.972  0.420060
  20  C5    c3    M   16  13  10     1.514   103.695  -111.946  0.257530
  21  H20   h1    E   20  16  13     1.090   113.314  -151.801  0.006070
  22  F21   f     E   20  16  13     1.380   110.438    87.062 -0.238040
  23  C6    c3    M   20  16  13     1.522   102.231   -30.070  0.399140
  24  H22   h2    E   23  20  16     1.093   112.129   -75.233  0.020130
  25  N8    na    M   23  20  16     1.460   112.802   162.332 -0.147810
  26  C9    cc    M   25  23  20     1.370   127.533   -82.906 -0.227610
  27  H23   h4    E   26  25  23     1.084   122.222    -0.013  0.176680
  28  C10   cd    M   26  25  23     1.386   104.733   179.986  0.047320
  29  H24   h4    E   28  26  25     1.085   126.008  -179.997  0.127130
  30  N11   nd    M   28  26  25     1.325   111.679    -0.029 -0.529330
  31  C12   cc    M   30  28  26     1.313   105.418     0.040  0.223370
  32  H25   h5    E   31  30  28     1.085   127.386  -179.959  0.084130


LOOP
   C6   O7
  C12   N8

IMPROPER
   C6   C9   N8  C12
  C10  H23   C9   N8
   C9  H24  C10  N11
  H25   N8  C12  N11

DONE
STOP
```

Table S22. prepi file for test molecule **19**.

```
    0    0    2

prepi file for test molecule 19
molecule.res
d19   INT  0
CORRECT     OMIT DU   BEG
  0.0000
   1  DUMM  DU    M    0  -1  -2     0.000      .0        .0      .00000
   2  DUMM  DU    M    1   0  -1     1.449      .0        .0      .00000
   3  DUMM  DU    M    2   1   0     1.523   111.21       .0      .00000
   4  C14   c3    M    3   2   1     1.540   111.208  -180.000 -0.098620
   5  H26   h1    E    4   3   2     1.090     0.000   -90.000  0.072560
   6  H27   h1    E    4   3   2     1.090   109.453  -120.008  0.077570
   7  H28   h1    E    4   3   2     1.090   109.453   120.008  0.080250
   8  N1    n3    M    4   3   2     1.460   109.490     0.000 -0.568160
   9  H15   hn    E    8   4   3     1.010   109.505   -59.943  0.311270
  10  C2    c3    M    8   4   3     1.460   109.490   180.000  0.041280
  11  H16   h1    E   10   8   4     1.090   109.453    59.992  0.044960
  12  H17   h1    E   10   8   4     1.090   109.453   -59.992  0.067350
  13  C3    c3    M   10   8   4     1.500   109.474  -180.000  0.180230
  14  O7    os    E   13  10   8     1.436   109.705    59.998 -0.372750
  15  H18   h1    E   13  10   8     1.092   107.677   -60.925  0.025630
  16  C4    c3    M   13  10   8     1.535   111.369   175.869  0.255960
  17  F13   f     E   16  13  10     1.379   109.041   133.545 -0.230040
  18  H19   h1    E   16  13  10     1.091   114.423    13.693  0.012770
  19  C5    c3    M   16  13  10     1.514   103.695  -111.946  0.065320
  20  O20   oh    S   19  16  13     1.410   113.305  -151.801 -0.533510
  21  H29   ho    E   20  19  16     0.961   109.496   179.992  0.386950
  22  H21   h1    E   19  16  13     1.091   110.440    87.060  0.086480
  23  C6    c3    M   19  16  13     1.522   102.231   -30.070  0.128820
  24  H22   h2    E   23  19  16     1.093   112.129   -75.233  0.124500
  25  N8    na    M   23  19  16     1.460   112.802   162.332  0.019780
  26  C9    cc    M   25  23  19     1.370   127.533   -82.906 -0.321120
  27  H23   h4    E   26  25  23     1.084   122.222    -0.013  0.197030
  28  C10   cd    M   26  25  23     1.386   104.733   179.986  0.094920
  29  H24   h4    E   28  26  25     1.085   126.008  -179.997  0.117740
  30  N11   nd    M   28  26  25     1.325   111.679    -0.029 -0.523300
  31  C12   cc    M   30  28  26     1.313   105.418     0.040  0.151500
  32  H25   h5    E   31  30  28     1.085   127.386  -179.959  0.104630


LOOP
   C6   O7
  C12   N8

IMPROPER
   C6   C9   N8  C12
  C10  H23   C9   N8
   C9  H24  C10  N11
  H25   N8  C12  N11

DONE
STOP
```

Table S23. prepi file for test molecule **20**.

```
    0    0    2

prepi file for test molecule 20
molecule.res
d20   INT  0
CORRECT     OMIT DU   BEG
  0.0000
   1  DUMM  DU    M    0  -1  -2     0.000      .0        .0      .00000
   2  DUMM  DU    M    1   0  -1     1.449      .0        .0      .00000
   3  DUMM  DU    M    2   1   0     1.523   111.21       .0      .00000
   4  C14   c3    M    3   2   1     1.540   111.208  -180.000 -0.024990
   5  H26   h1    E    4   3   2     1.090     0.000   -90.000  0.059100
   6  H27   h1    E    4   3   2     1.090   109.453  -120.008  0.055090
   7  H28   h1    E    4   3   2     1.090   109.453   120.008  0.061110
   8  N1    n3    M    4   3   2     1.460   109.490     0.000 -0.573530
   9  H15   hn    E    8   4   3     1.010   109.505   -59.943  0.311200
  10  C2    c3    M    8   4   3     1.460   109.490   180.000 -0.008210
  11  H16   h1    E   10   8   4     1.090   109.453    59.992  0.076970
  12  H17   h1    E   10   8   4     1.090   109.453   -59.992  0.075620
  13  C3    c3    M   10   8   4     1.500   109.474  -180.000  0.094110
  14  O7    os    E   13  10   8     1.436   109.705    59.998 -0.344560
  15  H18   h1    E   13  10   8     1.092   107.677   -60.925  0.064140
  16  C4    c3    M   13  10   8     1.535   111.369   175.869  0.162310
  17  O13   oh    S   16  13  10     1.410   109.046   133.545 -0.541810
  18  H29   ho    E   17  16  13     0.960   109.464  -179.983  0.379370
  19  H19   h1    E   16  13  10     1.091   114.423    13.693  0.045970
  20  C5    c3    M   16  13  10     1.514   103.695  -111.946  0.183920
  21  F20   f     E   20  16  13     1.380   113.304  -151.791 -0.223740
  22  H21   h1    E   20  16  13     1.091   110.440    87.060  0.046600
  23  C6    c3    M   20  16  13     1.522   102.231   -30.070  0.164570
  24  H22   h2    E   23  20  16     1.093   112.129   -75.233  0.097240
  25  N8    na    M   23  20  16     1.460   112.802   162.332  0.037240
  26  C9    cc    M   25  23  20     1.370   127.533   -82.906 -0.325790
  27  H23   h4    E   26  25  23     1.084   122.222    -0.013  0.195390
  28  C10   cd    M   26  25  23     1.386   104.733   179.986  0.111640
  29  H24   h4    E   28  26  25     1.085   126.008  -179.997  0.111200
  30  N11   nd    M   28  26  25     1.325   111.679    -0.029 -0.522210
  31  C12   cc    M   30  28  26     1.313   105.418     0.040  0.110300
  32  H25   h5    E   31  30  28     1.085   127.386  -179.959  0.121750


LOOP
   C6   O7
  C12   N8

IMPROPER
   C6   C9   N8  C12
  C10  H23   C9   N8
   C9  H24  C10  N11
  H25   N8  C12  N11

DONE
STOP
```

Table S24. prepi file for test molecule **21**.

```
    0    0    2

prepi file for test molecule 21
molecule.res
d21   INT  0
CORRECT     OMIT DU   BEG
  0.0000
   1  DUMM  DU    M    0  -1  -2     0.000      .0        .0      .00000
   2  DUMM  DU    M    1   0  -1     1.449      .0        .0      .00000
   3  DUMM  DU    M    2   1   0     1.523   111.21       .0      .00000
   4  C14   c3    M    3   2   1     1.540   111.208  -180.000 -0.053740
   5  H26   h1    E    4   3   2     1.090     0.000   -90.000  0.065380
   6  H27   h1    E    4   3   2     1.090   109.453  -120.008  0.061910
   7  H28   h1    E    4   3   2     1.090   109.453   120.008  0.070440
   8  N1    n3    M    4   3   2     1.460   109.490     0.000 -0.566820
   9  H15   hn    E    8   4   3     1.010   109.505   -59.943  0.311590
  10  C2    c3    M    8   4   3     1.460   109.490   180.000 -0.039580
  11  H16   h1    E   10   8   4     1.090   109.453    59.992  0.077430
  12  H17   h1    E   10   8   4     1.090   109.453   -59.992  0.085160
  13  C3    c3    M   10   8   4     1.500   109.474  -180.000  0.184330
  14  O7    os    E   13  10   8     1.436   109.705    59.998 -0.385120
  15  H18   h1    E   13  10   8     1.092   107.677   -60.925  0.039380
  16  C4    c3    M   13  10   8     1.535   111.369   175.869  0.184760
  17  H13   h1    E   16  13  10     1.089   109.031   133.558  0.064200
  18  F19   f     E   16  13  10     1.380   114.456    13.685 -0.232690
  19  C5    c3    M   16  13  10     1.514   103.695  -111.946  0.004860
  20  O20   oh    S   19  16  13     1.410   113.305  -151.801 -0.521200
  21  H29   ho    E   20  19  16     0.961   109.496   179.992  0.380450
  22  H21   h1    E   19  16  13     1.091   110.440    87.060  0.116060
  23  C6    c3    M   19  16  13     1.522   102.231   -30.070  0.279390
  24  H22   h2    E   23  19  16     1.093   112.129   -75.233  0.067810
  25  N8    na    M   23  19  16     1.460   112.802   162.332 -0.056770
  26  C9    cc    M   25  23  19     1.370   127.533   -82.906 -0.280480
  27  H23   h4    E   26  25  23     1.084   122.222    -0.013  0.193090
  28  C10   cd    M   26  25  23     1.386   104.733   179.986  0.068750
  29  H24   h4    E   28  26  25     1.085   126.008  -179.997  0.126030
  30  N11   nd    M   28  26  25     1.325   111.679    -0.029 -0.531790
  31  C12   cc    M   30  28  26     1.313   105.418     0.040  0.179020
  32  H25   h5    E   31  30  28     1.085   127.386  -179.959  0.108150


LOOP
   C6   O7
  C12   N8

IMPROPER
   C6    C9    N8  C12
  C10   H23    C9   N8
   C9   H24   C10  N11
  H25    N8   C12  N11

DONE
STOP
```

Table S25. prepi file for test molecule **22**.

```
    0    0    2

prepi file for test molecule 22
molecule.res
d22   INT  0
CORRECT     OMIT DU   BEG
  0.0000
   1  DUMM  DU    M    0  -1  -2     0.000      .0        .0      .00000
   2  DUMM  DU    M    1   0  -1     1.449      .0        .0      .00000
   3  DUMM  DU    M    2   1   0     1.523   111.21       .0      .00000
   4  C14   c3    M    3   2   1     1.540   111.208  -180.000 -0.018020
   5  H26   h1    E    4   3   2     1.090     0.000   -90.000  0.059800
   6  H27   h1    E    4   3   2     1.090   109.453  -120.008  0.060760
   7  H28   h1    E    4   3   2     1.090   109.453   120.008  0.062640
   8  N1    n3    M    4   3   2     1.460   109.490     0.000 -0.632340
   9  H15   hn    E    8   4   3     1.010   109.505   -59.943  0.316860
  10  C2    c3    M    8   4   3     1.460   109.490   180.000  0.056220
  11  H16   h1    E   10   8   4     1.090   109.453    59.992  0.055580
  12  H17   h1    E   10   8   4     1.090   109.453   -59.992  0.059410
  13  C3    c3    M   10   8   4     1.500   109.474  -180.000  0.236910
  14  O7    os    E   13  10   8     1.436   109.705    59.998 -0.408790
  15  H18   h1    E   13  10   8     1.092   107.677   -60.925  0.003040
  16  C4    c3    M   13  10   8     1.535   111.369   175.869  0.166520
  17  H13   h1    E   16  13  10     1.089   109.031   133.558  0.031130
  18  O19   oh    S   16  13  10     1.409   114.435    13.689 -0.600600
  19  H29   ho    E   18  16  13     0.960   109.498  -179.972  0.402130
  20  C5    c3    M   16  13  10     1.514   103.695  -111.946  0.227530
  21  F20   f     E   20  16  13     1.380   113.304  -151.791 -0.253740
  22  H21   h1    E   20  16  13     1.091   110.440    87.060  0.037790
  23  C6    c3    M   20  16  13     1.522   102.231   -30.070  0.286240
  24  H22   h2    E   23  20  16     1.093   112.129   -75.233  0.058660
  25  N8    na    M   23  20  16     1.460   112.802   162.332 -0.090390
  26  C9    cc    M   25  23  20     1.370   127.533   -82.906 -0.222120
  27  H23   h4    E   26  25  23     1.084   122.222    -0.013  0.171410
  28  C10   cd    M   26  25  23     1.386   104.733   179.986  0.065750
  29  H24   h4    E   28  26  25     1.085   126.008  -179.997  0.118210
  30  N11   nd    M   28  26  25     1.325   111.679    -0.029 -0.524270
  31  C12   cc    M   30  28  26     1.313   105.418     0.040  0.165950
  32  H25   h5    E   31  30  28     1.085   127.386  -179.959  0.107730


LOOP
   C6   O7
  C12   N8

IMPROPER
   C6   C9   N8  C12
  C10  H23   C9   N8
   C9  H24  C10  N11
  H25   N8  C12  N11

DONE
STOP
```

Table S26. prepi file for test molecule **23**.

```
    0    0    2

prepi file for test molecule 23
molecule.res
d23   INT  0
CORRECT     OMIT DU   BEG
  0.0000
   1  DUMM  DU    M    0  -1  -2     0.000      .0        .0      .00000
   2  DUMM  DU    M    1   0  -1     1.449      .0        .0      .00000
   3  DUMM  DU    M    2   1   0     1.523   111.21       .0      .00000
   4  C14   c3    M    3   2   1     1.540   111.208  -180.000  0.025640
   5  H26   h1    E    4   3   2     1.090     0.000   -90.000  0.040870
   6  H27   h1    E    4   3   2     1.090   109.453  -120.008  0.046220
   7  H28   h1    E    4   3   2     1.090   109.453   120.008  0.055500
   8  N1    n3    M    4   3   2     1.460   109.490     0.000 -0.609000
   9  H15   hn    E    8   4   3     1.010   109.505   -59.943  0.307900
  10  C2    c3    M    8   4   3     1.460   109.490   180.000  0.064400
  11  H16   h1    E   10   8   4     1.090   109.453    59.992  0.038480
  12  H17   h1    E   10   8   4     1.090   109.453   -59.992  0.057870
  13  C3    c3    M   10   8   4     1.500   109.474  -180.000  0.143610
  14  O7    os    E   13  10   8     1.436   109.705    59.998 -0.380390
  15  H18   h1    E   13  10   8     1.092   107.677   -60.925  0.038270
  16  C4    c3    M   13  10   8     1.535   111.369   175.869  0.338720
  17  F13   f     E   16  13  10     1.379   109.041   133.545 -0.273350
  18  H19   h1    E   16  13  10     1.091   114.423    13.693  0.029850
  19  C5    c3    M   16  13  10     1.514   103.695  -111.946 -0.034370
  20  H20   h1    E   19  16  13     1.090   113.314  -151.801  0.099480
  21  O21   oh    S   19  16  13     1.410   110.427    87.045 -0.553710
  22  H29   ho    E   21  19  16     0.961   109.504   179.965  0.414420
  23  C6    c3    M   19  16  13     1.522   102.231   -30.070  0.238710
  24  H22   h2    E   23  19  16     1.093   112.129   -75.233  0.070440
  25  N8    na    M   23  19  16     1.460   112.802   162.332 -0.032620
  26  C9    cc    M   25  23  19     1.370   127.533   -82.906 -0.226600
  27  H23   h4    E   26  25  23     1.084   122.222    -0.013  0.169960
  28  C10   cd    M   26  25  23     1.386   104.733   179.986  0.056060
  29  H24   h4    E   28  26  25     1.085   126.008  -179.997  0.123590
  30  N11   nd    M   28  26  25     1.325   111.679    -0.029 -0.519890
  31  C12   cc    M   30  28  26     1.313   105.418     0.040  0.151750
  32  H25   h5    E   31  30  28     1.085   127.386  -179.959  0.118190


LOOP
   C6   O7
  C12   N8

IMPROPER
   C6   C9   N8  C12
  C10  H23   C9   N8
   C9  H24  C10  N11
  H25   N8  C12  N11

DONE
STOP
```

Table S27. prepi file for test molecule **24**.

```
    0    0    2

prepi file for test molecule 24
molecule.res
d24   INT  0
CORRECT     OMIT DU   BEG
  0.0000
   1  DUMM  DU    M    0  -1  -2     0.000      .0        .0      .00000
   2  DUMM  DU    M    1   0  -1     1.449      .0        .0      .00000
   3  DUMM  DU    M    2   1   0     1.523   111.21       .0      .00000
   4  C14   c3    M    3   2   1     1.540   111.208  -180.000 -0.038480
   5  H26   h1    E    4   3   2     1.090     0.000   -90.000  0.059410
   6  H27   h1    E    4   3   2     1.090   109.453  -120.008  0.063560
   7  H28   h1    E    4   3   2     1.090   109.453   120.008  0.057950
   8  N1    n3    M    4   3   2     1.460   109.490     0.000 -0.570780
   9  H15   hn    E    8   4   3     1.010   109.505   -59.943  0.296900
  10  C2    c3    M    8   4   3     1.460   109.490   180.000  0.093700
  11  H16   h1    E   10   8   4     1.090   109.453    59.992  0.041300
  12  H17   h1    E   10   8   4     1.090   109.453   -59.992  0.042380
  13  C3    c3    M   10   8   4     1.500   109.474  -180.000  0.109590
  14  O7    os    E   13  10   8     1.436   109.705    59.998 -0.382410
  15  H18   h1    E   13  10   8     1.092   107.677   -60.925  0.051090
  16  C4    c3    M   13  10   8     1.535   111.369   175.869  0.149070
  17  O13   oh    S   16  13  10     1.410   109.046   133.545 -0.555110
  18  H29   ho    E   17  16  13     0.960   109.464  -179.983  0.383930
  19  H19   h1    E   16  13  10     1.091   114.423    13.693  0.072950
  20  C5    c3    M   16  13  10     1.514   103.695  -111.946  0.025540
  21  H20   h1    E   20  16  13     1.090   113.314  -151.801  0.093680
  22  F21   f     E   20  16  13     1.380   110.438    87.062 -0.188880
  23  C6    c3    M   20  16  13     1.522   102.231   -30.070  0.370790
  24  H22   h2    E   23  20  16     1.093   112.129   -75.233  0.045220
  25  N8    na    M   23  20  16     1.460   112.802   162.332 -0.126130
  26  C9    cc    M   25  23  20     1.370   127.533   -82.906 -0.234750
  27  H23   h4    E   26  25  23     1.084   122.222    -0.013  0.181040
  28  C10   cd    M   26  25  23     1.386   104.733   179.986  0.049070
  29  H24   h4    E   28  26  25     1.085   126.008  -179.997  0.132920
  30  N11   nd    M   28  26  25     1.325   111.679    -0.029 -0.537190
  31  C12   cc    M   30  28  26     1.313   105.418     0.040  0.216600
  32  H25   h5    E   31  30  28     1.085   127.386  -179.959  0.097040


LOOP
   C6   O7
  C12   N8

IMPROPER
   C6   C9   N8  C12
  C10  H23   C9   N8
   C9  H24  C10  N11
  H25   N8  C12  N11

DONE
STOP
```
